# Divergent projections of the prelimbic cortex mediate autism- and anxiety-like behaviors

**DOI:** 10.1038/s41380-023-01954-y

**Published:** 2023-01-23

**Authors:** Yi-Fan Luo, Lu Lu, Heng-Yi Song, Han Xu, Zhi-Wei Zheng, Zhou-Yue Wu, Chen-Chen Jiang, Chu Tong, Hao-Yang Yuan, Xiu-Xiu Liu, Xiang Chen, Mei-ling Sun, Ya-Min Tang, Heng-Yu Fan, Feng Han, Ying-Mei Lu

**Affiliations:** 1 International Joint Laboratory for Drug Target of Critical Illnesses, Key Laboratory of Cardiovascular and Cerebrovascular Medicine, School of Pharmacy, Nanjing Medical University, Nanjing, 211166 China; 2 Department of Physiology, School of Basic Medical Sciences, Nanjing Medical University, Nanjing, 211166 China; 3 Life Sciences Institute and Innovation Center for Cell Biology, Zhejiang University, Hangzhou, 310058 China; 4 Institute of Brain Science, the Affiliated Brain Hospital of Nanjing Medical University, Nanjing, 211166 China

**Keywords:** Neuroscience, Drug discovery

## Abstract

The comorbidity of autism spectrum disorder and anxiety is common, but the underlying circuitry is poorly understood. Here, *Tmem74*^*-/-*^ mice showed autism- and anxiety-like behaviors along with increased excitability of pyramidal neurons (PNs) in the prelimbic cortex (PL), which were reversed by *Tmem74* re-expression and chemogenetic inhibition in PNs of the PL. To determine the underlying circuitry, we performed conditional deletion of *Tmem74* in the PNs of PL of mice, and we found that alterations in the PL projections to fast-spiking interneurons (FSIs) in the dorsal striatum (dSTR) (PL^PNs^–dSTR^FSIs^) mediated the hyperexcitability of FSIs and autism-like behaviors and that alterations in the PL projections to the PNs of the basolateral amygdaloid nucleus (BLA) (PL^PNs^–BLA^PNs^) mediated the hyperexcitability of PNs and anxiety-like behaviors. However, the two populations of PNs in the PL had different spatial locations, optogenetic manipulations revealed that alterations in the activity in the PL–dSTR or PL–BLA circuits led to autism- or anxiety-like behaviors, respectively. Collectively, these findings highlight that the hyperactivity of the two populations of PNs in the PL mediates autism and anxiety comorbidity through the PL–dSTR and PL–BLA circuits, which may lead to the development of new therapeutics for the autism and anxiety comorbidity.

## Introduction

Autism spectrum disorder (ASD) is a common neuropsychiatric disorder characterized by the presence of stereotyped behaviors and deficits in social communication skills. The genetic abnormalities associated with ASD are single or polygenic risk factors caused by variants [1, 2]. Given the enormous heterogeneity in the manifestations of the core features of ASD [3], genetic mutations in related diseases, such as mutations in *SHANK2* and *SHANK3* in autism, *MECP2* in Rett’s syndrome, and *FMR1* in fragile X syndrome, have become the focus of studies [4–7]. Most of the known genetic alterations contributing to ASD affect the expression or function of proteins with established roles in brain development and function [8].

As social behavior involves a range of cognitive processes, including attention, emotion, motivation, and memory [9], mutations in autism-related genes impact the functions of the medial prefrontal cortex (mPFC), which has long been considered a higher-order sensory cortex in generating social responses [10, 11]. The mPFC afferents to the anterior cingulate cortex (ACC), striatum, nucleus accumbens (NAc), BLA and so on, comprise the “social brain” and influence sociability [8, 12–15]. In particular, the subpopulations of PL (a subregion of the mPFC) neurons projecting to the NAc encode a conjunction of social and spatial information [16]. It has been reported that both elevated and reduced activation of the mPFC lead to social preference impairments [17, 18]. The pathway of the mPFC to the BLA is also implicated in ASD [19]. In addition, synaptic hyperconnectivity in the mPFC–BLA circuit occurs in response to social cues and social interaction deficits in heterozygous *Pten* mutant mice [20]. However, during emotional behavioral processes, the mPFC appears to exhibit division activities in different subregions, including the PL and infralimbic cortex (IL) [21–23]. Therefore, identifying the essential anatomical and functional substrates for PL or IL–BLA circuit is necessary to understand the neural mechanisms underlying emotional behavioral processes.

ASD and anxiety are highly comorbid conditions, and children with ASD have increased susceptibility to anxiety disorders [24, 25]. Researchers have assessed the increased risk of anxiety among individuals with ASD and found that they suffered from both common anxiety and ASD-related anxiety, which were less common in the nonautistic group [26]. Understanding the risk of comorbid mood and anxiety disorder is critical to improve the long-term treatment of ASD [27]. Thus, investigating the molecular mechanisms and circuits regulating the two comorbidities has great clinical guidance.

Transmembrane protein 74 (TMEM74) contains two transmembrane domains, and there is a high level of its corresponding mRNA in the brain [28]. However, studies on TMEM74 in the brain remain absent. Recently, we reported that the functional coupling of TMEM74 and hyperpolarization-activated cyclic nucleotide-gated 1 (HCN1) channels in the pyramidal neurons of the BLA of *Tmem74*^*-/-*^ mice regulated anxiety-like behaviors [29]. Understanding the dynamics of transmembrane proteins in the brain can accelerate the discovery of potential therapeutic targets for brain diseases.

In this study, our bioinformatics data validated the high correlation between *Tmem74* and ASD. We observed repetitive behaviors and social novelty preference impairments, together with cooccurring anxiety behaviors in *Tmem74*^*-/-*^ mice, accompanied by the activation of c-Fos in the PNs of the PL. Using anatomical and electrophysiological circuit-mapping techniques, we showed that divergent projections of the PL to dSTR (PL^PNs^–dSTR^FSIs^) and BLA (PL^PNs^–BLA^PNs^) mediate autism-anxiety comorbidity. Combining these experiments with optogenetic and pharmacological strategy revealed that TMEM74 in PL plays a critical role in the association with autism- and anxiety-like behaviors.

## Materials and methods

Animals, virus vectors, Stereotaxic injection, optical fiber and electrode implantation, behavioral tests, brain slice preparation, whole-cell recordings, drug treatment, western blotting, immunohistochemistry, identification of differentially expressed (DE) genes, and quantification and statistical analyses are described in detail in the supplementary materials and methods.

## Results

### *Tmem74*^*-/-*^ mice displayed stereotyped behaviors and social deficits, along with anxiety-like behaviors

**Fig. 1:**
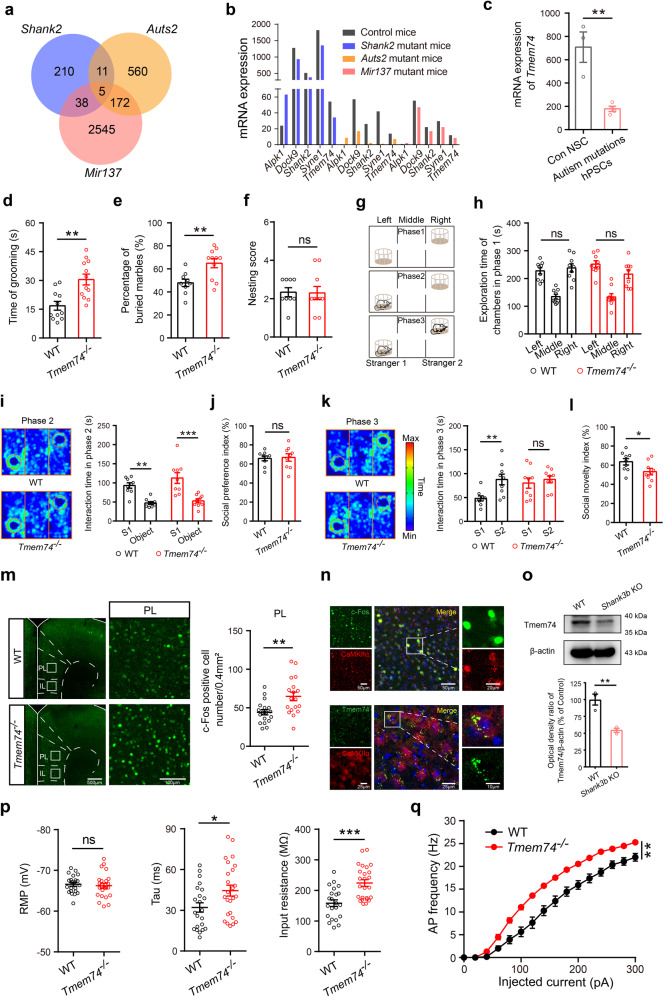
*Tmem74*^−/−^ mice display autistic-like behaviors and exhibit abnormal electrophysiological properties in PL pyramidal neurons. **a** An overlapping Venn diagram of transcriptomic differential genes in three types of mice with autistic-like behaviors. **b** The mRNA expression of 5 overlapping differentially expressed genes. **c** The mRNA expression of *Tmem74* in human pluripotent stem cells (hPSCs). **d** Quantification of the time in self-grooming during the 10-min free movement (*n*  =  11 WT mice, *n*  =  12 *Tmem74*^−/−^ mice). **e** The percentage of buried marbles (*n* =  9 WT mice, *n*  =  11 *Tmem74*^−/−^ mice). **f** The nesting ability (*n*  =  9 WT mice, *n*  =  10 *Tmem74*^−/−^ mice). **g** Schematic of the three-chamber social test. **h** The mice preference for either the left or right chamber during phase 1 (*n*  =  9 WT mice, *n*  =  9 *Tmem74*^−/−^ mice). **i** Left: Representative heatmaps of trajectory in phase 2 of three-chamber tests. Right: The time of interacting with the S1 mouse and the empty object (*n*  =  9 WT mice, *n*  =  9 *Tmem74*^−/−^ mice); S1: Strange mouse 1. **j** The social interaction preference index (*n*  = 9 WT mice, *n*  =  9 *Tmem74*^−/−^ mice). **k** Left: Representative heatmaps of trajectory in phase 3 of three-chamber tests. Right: The interaction time with S2 and S1 mouse (*n*  =  9 WT mice, *n*  =  9 *Tmem74*^−/−^ mice); S2: Strange mouse 2. **l** The social novelty index (*n*  =  9 WT mice, *n*  =  9 *Tmem74*^−/−^ mice). **m** Left: Representative confocal images of the c-Fos (green) after social interaction in the subregion of mPFC (PL). Right: Quantitative results of c-Fos activation in PL (*n*  =  3 in each group, and six views of slices were provided for each mouse). **n** Top: Representative images of immunofluorescence showed that c-Fos co-localized at the CaMKIIα-positive neurons in PL. Bottom: Representative images of co-localization of TMEM74 and CaMKIIα. **o** The protein level of TMEM74 in PL of *Shank3b* knockout mice (*n* = 3 in each group). **p** Quantification of RMP (Left), Tau (Middle), and Rin (Right) of membrane properties in PL pyramidal neurons under whole-cell recording (*n*  =  22 cells from 7 WT mice, *n*  =  25 cells from 5 *Tmem74*^−/−^ mice). **q** Quantification of the AP frequency by current injections from 0 to 300  pA (stepped by 20 pA) (*n*  =  22 cells from 7 WT mice, *n*  =  25 cells from 5 *Tmem74*^−/−^ mice). Data were presented as means  ± SEM. *P  <  0.05, **P  <  0.01, ***P  <  0.001; ns not significant. Unpaired two-tailed Student’s *t*-test for **c**–**f**, **j**, **l**, **m**, **o** and **p**; Two-way ANOVA followed by Sidak’s post hoc test for **h**, **i** and **k**; Two-way ANOVA followed by Bonferroni’s post hoc test for **q**. mPFC medial prefrontal cortex, PL prelimbic cortex, RMP resting membrane potential, Tau time constant, Rin input resistant.

Since genetic analyses have linked *MIR137*, *SHANK2*, and *AUTS2* to ASD, we utilized transcriptomics profiling analysis in a database of three candidate gene mutant mice to find their intersection. Sequencing results of brain tissue samples from three ASD mutant mice revealed 5 overlapping differential genes (*Alpk1*, *Dock9*, *Shank2*, *Syne1, and Tmem74*) (Fig. 1a, b) [30–32]. Among the downregulated genes, *Dock9* was reported to be associated with peripheral cancers [33]. *Syne1* mutations have been shown to play important roles in cerebellar ataxia [34, 35]. *Shank2*, a multidomain scaffolding protein enriched at excitatory neuronal synapses, has been shown to regulate autism-like social behaviors [5, 36]. *Tmem74*, the gene of interest in this study, was distributed highly on neurons, and the mRNA expression of *Tmem74* showed a consistent downward trend in these three ASD mutant mice (Fig. 1b). Furthermore, from a multifunctional human stem cell platform database [37], we found that the mRNA level of *Tmem74* was also remarkably decreased in human pluripotent stem cells (hPSCs) with early autism-related mutation genes (Fig. 1c). Together, these results strongly suggested that *Tmem74* had a strong correlation with ASD and could be a novel autism-related gene.

To investigate the effect of *Tmem74* in ASD, we performed autism-like behavioral analyses in *Tmem74*^−/−^ mice. First, stereotyped and digging behavior tests were performed. Compared to WT mice, *Tmem74*^−/−^ mice showed increased repetitive grooming (Fig. 1d) and numbers of buried marbles (Fig. 1e) but no changes in the scores of the nesting experiment (Fig. 1f). Second, a three-chambered social novelty assay was performed (Fig. 1g). In the phase 1 test, the mice showed no left-right preference (Fig. 1h). In the phase 2 test, mice in both groups explored the first strange mouse 1 (S1) more time than the empty cage (Fig. 1i), which indicated no alteration in the social preferences of *Tmem74*^−/−^ mice (Fig. 1j). However, in the phase 3 test, *Tmem74*^−/−^ mice presented no social novelty preference for the new strange mouse 2 (S2) (Fig. 1k), and the social novelty index was reduced (Fig. 1l).

Next, we also examined the anxiety-like phenotype of *Tmem74*^−*/*−^ mice as previously described [29]. In the open field test (OFT) (Supplementary Fig. 1a), compared to WT mice, *Tmem74*^−*/*−^ mice spent less time in the central zone and more time in the surrounding zone (Supplementary Fig. 1b, d). However, the number of entries into the center zone and the total distance traveled were not changed in the two groups of mice (Supplementary Fig. 1c, e). In the elevated plus maze (EPM) analysis (Supplementary Fig. 1f), compared to WT mice, *Tmem74*^−*/*−^ mice exhibited shorter travel time, a decreased number of entries into the open arms, more time in the closed arms, and no change in the total distance traveled (Supplementary Fig. 1g-j). Finally, to determine whether the *Tmem74* deficit affected memory and learning, Y-maze and novel object exploration assays were performed. The data suggested that there were no changes in *Tmem74*^−*/*−^ mice or WT mice (Supplementary Fig. 1k–n).

Together, the data indicated that the *Tmem74* deficit induced autism- and anxiety-like behaviors, but did not affect learning and memory in mice.

### Increased membrane properties and excitability of PL pyramidal neurons in *Tmem74*^−*/*−^ mice

To identify the key brain region involved in repetitive and socially deficient behaviors, we examined the expression of the immediate early gene *c-fos* in the brain [38, 39]. Compared to WT mice, the expression of c-Fos in CaMKIIα-positive neurons was upregulated in the PL of *Tmem74*^−*/*−^ mice (Fig. 1m, n), while there was no change in the IL and ACC after social interaction (Supplementary Fig. 2a, b). Moreover, we also identified the downregulation of TMEM74 in the PL of *Shank3* knockout mice (Fig. 1o), implying that the *Tmem74* deficit in PL guides autism-like behaviors.

Next, to examine the effects of TMEM74 on the PNs of the PL, we confirmed the electrophysiological characteristics of PNs via whole-cell patch recording. The time constant (Tau) and input resistance (Rin) were both aggrandized, while the resting membrane potential (RMP) was unchanged in the PNs of the PLs of *Tmem74*^−*/*−^ mice (Fig. 1p). Moreover, PNs in the PL of *Tmem74*^−*/*−^ mice were more sensitive to depolarizing current injections with higher excitability than those of WT mice (Fig. 1q). However, the properties of action potentials (APs) showed unaltered parameters such as amplitude, release threshold, half width and after hyperpolarization potential (AHP) (Supplementary Fig. 2c–f).

### Chemogenetic manipulation of PL pyramidal neurons reversed stereotyped behaviors and social novelty deficits in *Tmem74*^*-/-*^ mice

**Fig. 2:**
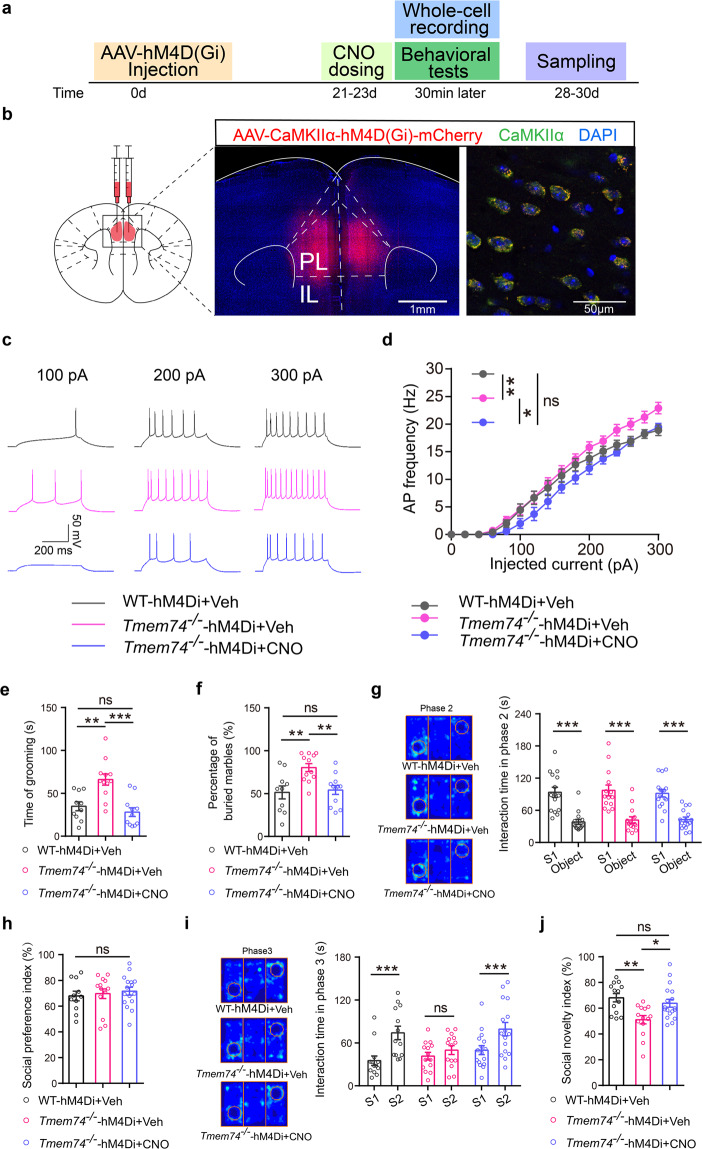
Chemogenetic inhibition of PL pyramidal neurons ameliorates repetitive behavior and social novelty impairment. **a** Diagram of virus injection and experimental procedure in WT and *Tmem74*^−*/*−^ mice. **b** The injecting site of pAAV2/9-CaMKIIα-hM4D(Gi)-mCherry-3FLAG (AAV-CaMKIIα-hM4D(Gi)-mCherry) and validation of hM4D(Gi)-mCherry in PL pyramidal neurons in *Tmem74*^−/−^ mice. **c** Representative spike firing of PL pyramidal neurons in hM4D(Gi) expression brain slices incubated with CNO (10 μmol/L). **d** Quantification of the AP frequency by current injections from 0 to 300 pA (stepped by 20 pA) (*n* = 9 cells from 3 WT- hM4Di+Veh mice, *n* = 11 cells from 3 *Tmem74*^−/−^-hM4Di+Veh mice, *n* = 7 cells from 3 *Tmem74*^−/−^-hM4Di+CNO mice). **e**, **f** The time spent on grooming (**e**) and the percentage of buried marbles (**f**) in mice (**e**, *n* = 10 WT-hM4Di+Veh mice, *n* = 12 *Tmem74*^−/−^-hM4Di+Veh mice, *n* = 11 *Tmem74*^−/−^-hM4Di+CNO mice; **f**, *n* = 10 WT-hM4Di+Veh mice, *n* = 12 *Tmem74*^−/−^-hM4Di+Veh mice, *n* = 12 *Tmem74*^−/−^-hM4Di+CNO mice). **g**–**j** The interaction time (**g**) and social preference index (**h**) in all groups during phase 2, and the interaction time (**i**) and social novelty index (**j**) during phase 3 from three-chamber test (*n* = 13 WT-hM4Di+Veh mice, *n* = 14 *Tmem74*^−/−^-hM4Di+Veh mice, *n* = 16 *Tmem74*^−/−^-hM4Di+CNO mice). Data were presented as means ± SEM. *P < 0.05, **P < 0.01, ***P < 0.001; ns not significant. Two-way ANOVA followed by Bonferroni’s post hoc test for **d**; One-way ANOVA followed by Tukey’s post hoc test for **e**, **f**; Two-way ANOVA followed by Bonferroni’s post hoc test for **g**; One-way ANOVA followed by Bonferroni’s post hoc test for **h**; Two-way ANOVA followed by Sidak’s post hoc test for **i**; one**-**way ANOVA followed by Sidak’s post hoc test for **j**.

To verify the hypothesis that the hyperexcitability of PNs in PL guides autism-like behaviors, we expressed AAV-CaMKIIα-hM4D(Gi)-mCherry in the PL of 4-week-old WT and *Tmem74*^−*/*−^ mice (Fig. 2a, b) under intraperitoneal (i.p.) injection of clozapine-N-oxide (CNO, 1 mg/kg) [40]. First, whole-cell patch recording data demonstrated that the elevation of APs in PNs of *Tmem74*^−*/*−^ mice was reversed under chemogenetic inhibition manipulation (Fig. 2c, d). Next, stereotyped behavior and three-chambered social novelty analysis were performed. Chemogenetic inhibition of PL pyramidal neurons in *Tmem74*^−*/*−^ mice dramatically reduced the grooming time and the number of buried marbles (Fig. 2e, f). For three-chamber social interaction tests, in the phase 2 test, the interaction time with the first strange mouse 1 (S1) did not differ among the three groups of mice (Fig. 2g), which indicated no alteration in the social preferences of mice (Fig. 2h). In the phase 3 test, *Tmem74*^−*/*−^ mice presented no social novelty preference for the new strange mouse 2 (S2), which was reversed by chemogenetic manipulation (Fig. 2i). Additionally, changes of social novelty index in *Tmem74*^−*/*−^ mice were reversed by chemogenetic manipulation (Fig. 2j).

Consistent with this hypothesis, the results demonstrated that the chemogenetic inhibition of PL pyramidal neurons ameliorated autistic-like behaviors in *Tmem74*^−/−^ mice, which suggested that *Tmem74*-induced hyperexcitability in the PL was responsible for autistic-like behaviors.

### Conditional deletion of *Tmem74* in the PNs of the PL caused repetitive behaviors and social novelty deficits, along with anxiety-like behaviors

**Fig. 3:**
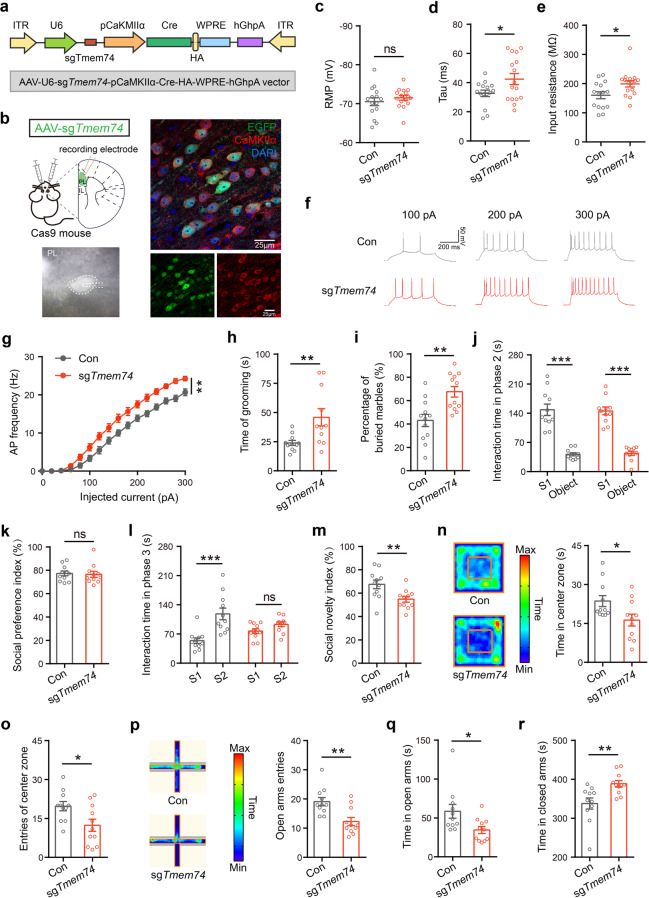
Selective deletion of *TMEM74* in PL leads to autism- and anxiety-like behaviors. **a** Schematic illustration of the AAV-sg*Tmem74* which relied on CaMKIIα promotor. **b** Left: Diagram of virus injection site and representative pyramidal neuron under whole-cell patch recording. Right: the colocation of EGFP- and CaMKII-positive neurons in PL region of Cre-dependent Rosa26-LSL-Cas9 mice (Cas9 mice) after pAAV2/9-U6-sg*Tmem74*-CaMKIIα-Cre-HA-WPRE-hGHpA (AAV-sg*Tmem74*) injection. DAPI for nuclei. **c**–**e** Membrane properties of pyramidal neurons in PL of control (Con) or AAV-sg*Tmem74*-injected mice (sg*Tmem74*), RMP (**c**), Tau (**d**) and Rin (**e**) (*n* = 15 cells from 4 control mice, *n* = 16 cells from 3 AAV-sg*Tmem74*-injected mice). **f** Representative AP firing of PL pyramidal neuron. **g** Quantification of AP frequency by current injections from 0 to 300 pA (stepped by 20 pA) (*n* = 15 cells from 4 control mice, n = 16 cells from 3 AAV-sg*Tmem74*-injected mice). **h**, **i** The time spent on grooming (**h**) and the percentage of buried marbles (**i**) in control mice and in selective loss of *Tmem74* mice (**h**, *n* = 11 in each group; **i**, *n* = 12 in each group). **j**–**m** The interaction time (**j**) and social preference index (**k**) in all groups during phase 2, and the interaction time (**l**) and social novelty index (**m**) during phase 3 from three-chamber test (*n* = 11 in each group). **n**, **o** Representative heatmaps and time in central zone (**n**), the entries of central zone (**o**) in the open field test (*n* = 11 in each group). **p**–**r** Representative heatmaps and entries of open arms in the elevated plus maze (**p**), the time in open arms (**q**) and the exploring time in closed arms (**r**) (*n* = 11 in each group). Data were presented as means ± SEM. *P < 0.05, **P < 0.01, ***P < 0.001; ns not significant. Unpaired two-tailed Student’s *t*-test for **c**–**e**, **h**, **i**, **k**, **m**–**r**; Two-way ANOVA followed by Bonferroni’s post hoc test for **g**; Two-way ANOVA followed by Sidak’s post hoc test for **j** and **l**.

To investigate the causal link between the PL regional *Tmem74* deficit in PNs and the autistic-like phenotype, we selectively decreased *Tmem74* expression in the PNs of the PL through a CRISPR/Cas9 system [29]. An AAV2/9 vector harboring a single-guide RNA (AAV-U6-sg*Tmem74-*pCaMKIIa-Cre-HA-pWPRE-hGhpA, AAV-sg*Tmem74*) was infused bilaterally into the PL of 4-week-old Cre-dependent Cas9 mice (Fig. 3a, b). AAV-U6-EGFP*-*pCaMKIIa-Cre-HA-pWPRE-hGhp (AAV-EGFP) was used as a control injection. Western blotting and immunohistochemical staining identified the lower expression of TMEM74 in the PNs of the PLs of sg*Tmem74*-injected mice (Supplementary Fig. 3a, b). Consistent with *Tmem74*^−/−^ mice, increased membrane properties (Tau and Rin) (Fig. 3c–e) and AP frequency (Fig. 3f, g) were observed in the PL pyramidal neurons of AAV-sg*Tmem74*-injected mice, and the properties of APs (amplitude, threshold, half width and AHP) were unaltered (Supplementary Fig. 3c–f).

For stereotyped behavior analysis, AAV-sg*Tmem74*-injected mice showed increased grooming time and numbers of buried marbles (Fig. 3h, i). In three-chamber social interaction tests, AAV-sg*Tmem74*-injected mice also had intact social preferences and spent more time interacting with S1 mice in phase 2 (Fig. 3j, k). However, in phase 3, compared with mice in the control group administered AAV-EGFP, AAV-sg*Tmem74*-injected mice displayed no social novelty preference for S2 mice and a decreased social novelty index (Fig. 3l, m). Moreover, we carried out anxiety-related behavior tests. In the OFT, PNs *Tmem74*-deficient mice showed significantly less center exploration time, fewer center square entries and more time spent in corners (Fig. 3n, o; Supplementary Fig. 3g). In the EPM test, AAV-sg*Tmem74*-injected mice showed fewer entries and less time spent in the open arms (Fig. 3p, q) and more time spent in the closed arms than control mice (Fig. 3r). There was no difference in the total distance traveled in the open field test, while PL *Tmem74* PN-deficient mice had shorter total distances traveled in the elevated plus maze (Supplementary Fig. 3h, i).

These data confirmed that *Tmem74* deficiency in the PNs of the PL was sufficient to induce autism- and anxiety-like behaviors.

### PL–dSTR circuit medium spiny neurons mediated autistic-like behaviors

**Fig. 4:**
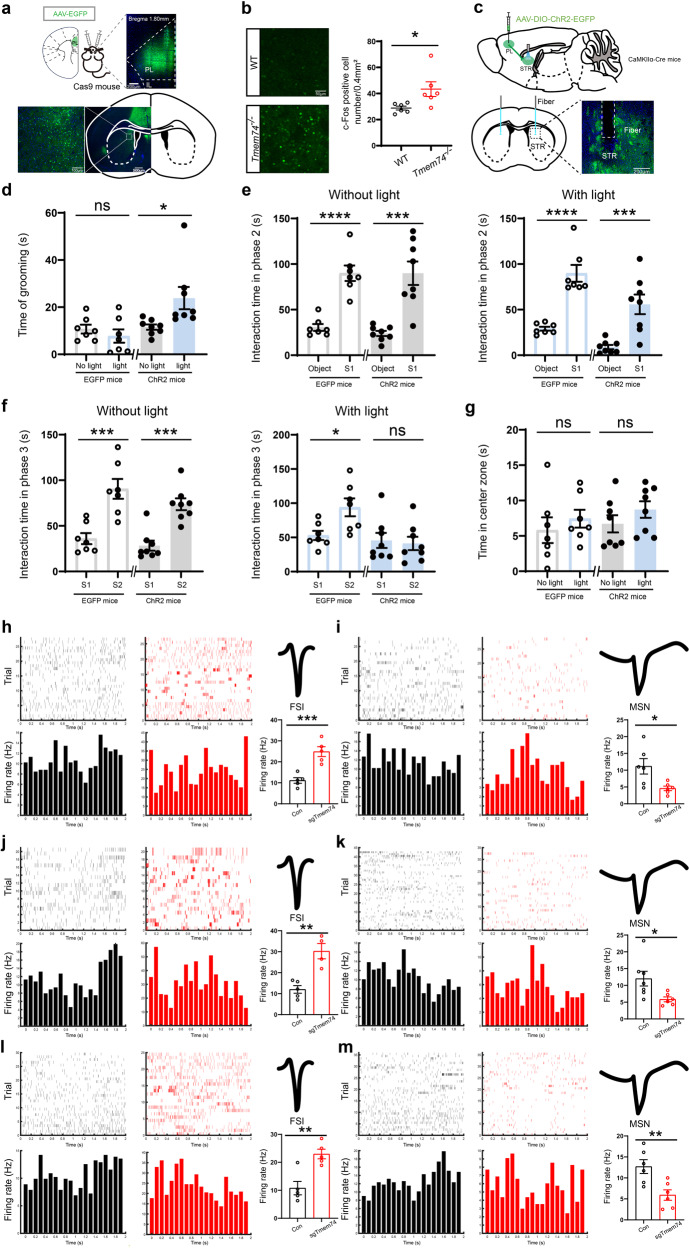
PL projecting to dSTR medium spiny neurons mediates autistic behaviors. **a** Diagram of pAAV2/9-CaMKIIα-EGFP-2A-MSC-3FLAG (AAV-EGFP) injection and the images of EGFP in PL region of Cas9 mice. **b** Representative confocal images and quantitative results of the c-Fos (green) after social interaction in the dSTR (*n* = 3 in each group, and two views of slices were provided for each mouse). **c** Schematic and histology of pAAV2/9-EF1α-DIO-EGFP-WPRE (AAV-DIO-EGFP) or pAAV2/9-EF1α-DIO-hChR2(H134R)-EGFP-WPRE (AAV-DIO-ChR2-EGFP) injection in PL and optical fiber placement targeting dSTR terminals. **d**–**g** Optogenetic activation of PL-dSTR led to autistic-like behavior in mice. CaMKIIα-Cre mice+AAV-DIO-EGFP: *n* = 7; CaMKIIα-Cre mice+AAV-DIO-ChR2-EGFP: *n* = 8. **d** The time of grooming with or without blue light stimulation. **e**, **f** The interaction time in three-chamber test during phase 2 (**e**) and phase 3 (**f**) with or without blue light stimulation. **g** Quantitation of the time in center zone in open field test with or without blue light stimulation. **h**–**m** Spike raster (Top) and PSTH (Bottom) of identified FSIs, waveform of identified FSIs (Top), and Quantitative results of firing rate of identified FSIs in grooming test (**h**, *n* = 5 units from 3 control mice, *n* = 5 units from 3 AAV-*sgTmem74*-injected mice), home-cage test (**j**, *n* = 5 units from 3 control mice, *n* = 4 units from 3 AAV-*sgTmem74*-injected mice) and three-chamber test (**l**, *n* = 5 units from 3 control mice, *n* = 5 units from 3 AAV-*sgTmem74*-injected mice); Spike raster (Top) and PSTH (Bottom) of identified MSNs, Waveform of identified MSNs (Top) and Quantitative results of firing rate of identified MSNs in grooming test (**i**, *n* = 6 units from 3 control mice, *n* = 6 units from 3 AAV-sg*Tmem74*-injected mice), home-cage test (**k**, *n* = 7 units from 3 control mice, *n* = 6 units from 3 AAV-sg*Tmem74*-injected mice) and three-chamber test (**m**, *n* = 6 units from 3 control mice, *n* = 6 units from 3 AAV-sg*Tmem74*-injected mice). Black column: control mice; Red column: AAV-sg*Tmem74* injected mice. Data were presented as means ± SEM. *P < 0.05, **P < 0.01, ***P < 0.001; ****P < 0.0001; ns not significant. dSTR: dorsal striatum, FSIs fast-spiking interneurons, MSNs medium spiny neurons. Unpaired two-tailed Student’s *t*-test for **b**, **e**, **f** and **h**–**m;** Paired two-tailed Student’s *t*-test for **d** and **g**.

To investigate the underlying circuitry of PL-mediated autism- and anxiety-like behaviors, PLs were injected with pAAV2/9-CaMKIIα-EGFP-2A-MSC-3FLAG (pAAV2/9-CaMKIIα-EGFP) (Fig. 4a). Green fluorescence was enriched in the dSTR (Fig. 4a). Moreover, we examined the expression of c-Fos in the dSTR after three-chamber social interaction. Compared to WT mice, the expression of c-Fos was increased in *Tmem74*^*-/-*^ mice (Fig. 4b).

To further confirm that the PL–dSTR circuit mediates autistic-like behaviors, we transiently activated PN projections to the STR (PL^PNs^–STR) through ChR2 optogenetic regulation (Fig. 4c, top). After AAV-EF1α-DIO-ChR2-EGFP (AAV-DIO-ChR2-EGFP) or AAV-EF1α-DIO-EGFP (AAV-DIO-EGFP) was injected into the PL of CaMKIIα-Cre mice, optic fibers were implanted into the dSTR of those mice (Fig. 4c, bottom). In the grooming test, optogenetic activation of PL–dSTR neurons terminals resulted in a significant increase in the grooming time (Fig. 4d). During the three-chamber test, PL–dSTR circuit activation resulted in social novelty deficits during phase 3 but not social preference deficits during phase2 (Fig. 4e, f), which is consistent with findings in *Tmem74*^−/−^ mice or AAV-sg*Tmem74*-injected mice. In addition, we conducted an open-field test to evaluate anxiety-like behaviors. The data showed that PL–dSTR circuit activation induced no changes in the two groups (Fig. 4g), which indicated that PL–dSTR circuit activation did not participate in anxiety-like behavior.

Next, to selectively examine the contribution of PL–dSTR circuit for ASD-like behaviors, we optogenetically suppressed PL^PNs^–STR and assessed the related behaviors in *Tmem74*^−/−^mice (Supplementary Fig. 4a). During the phase 3 of three-chamber test, optogenetic suppression of PL–dSTR circuit rescued social novelty deficits without impacting the social preference (Supplementary Fig. 4b, c). Consistent with this finding, we observed PL–dSTR suppression resulted in a significant decrease in the grooming test (Supplementary Fig. 4d). In addition, the data of open field test showed that PL–dSTR circuit suppression had no changes between the two groups (Supplementary Fig. 4e), which further strengthened the point that PL–dSTR circuit did not participate in anxiety-like behaviors.

To clarify the effects of PL–dSTR circuit activation on dSTR neurons, we conducted in vivo electrophysiological recordings by implanting electrodes into the dSTR of Cas9 and sg*Tmem74* mice. According to the obviously divergent waveform, we identified fast-spiking interneurons (FSIs) and medium spiny neurons (MSNs) [41] and then conducted further analyses on these two types of neurons. In accordance with the enhancing activity of PNs from the PL, the FSIs of sg*Tmem74* mice manifested a significantly higher firing rate than those of Cas9 control mice in the grooming test (Fig. 4h), home-cage test (Fig. 4j) and three-chamber test (Fig. 4l). Interestingly, MSNs, the main projection neuron in the dSTR, exhibited a significant decrease in firing rate in the grooming test (Fig. 4i), home-cage test (Fig. 4k) and three-chamber test (Fig. 4m), which was in total contrast to the increased activity of PNs from the PL. Considering the well-studied microcircuit between FSIs and MSNs in dSTR, where FSIs inhibited surrounding MSNs when the cortex was excited [42], we deduced that the increased FSI activation by glutamatergic projections from the PL influenced the surrounding MSNs through the FSI–MSN microcircuit of the dSTR.

These data suggested that excitatory hyperactivity of the PL–dSTR circuit resulted in an abnormal firing rate of FSIs and MSNs, thereby leading to autism-like behavior.

### PL–BLA circuit pyramidal neurons mediated anxiety-like behavior

In addition to the dSTR, fibers with obvious green fluorescence from the PL were also observed around the neuronal soma in the BLA as the PL was injected with pAAV2/9-CaMKIIα-EGFP (Supplementary Fig. 5a, b). Moreover, we found that a substantial number of EGFP-positive neurons in the BLA were colocalized with CaMKIIα-positive neurons (Supplementary Fig. 5c). Our previous study reported that selective deletion of *Tmem74* in the PNs of the BLA produced anxiety-like behaviors by elevating excitability of PNs in the BLA [29]. To verify that the circuit of PL^PNs^–BLA^PNs^ regulates the excitability of PNs in the BLA of sg*Tmem74* mice, we examined the electrophysiological characteristics of PNs in BLA by whole-cell patch recording while keeping (slicing scheme 1) or cutting off (slicing scheme 2) the PL–BLA circuit in brain slices (Supplementary Fig. 5d). The PNs of the BLA showed no changes in RMP, Tau, or Rin in slicing scheme 1 or 2 (Supplementary Fig. 5e, h). However, the excitability of PNs of the BLA became significantly higher with the increased AP frequency in slicing scheme 1 but not in slicing scheme 2 (Supplementary Fig. 5f, i). The AP properties, such as the amplitude, threshold, half width or after hyperpolarization potential (AHP), did not change in slicing schemes 1 or 2 (Supplementary Fig. 5g, j).

To further confirm that the PL–BLA circuit mediates anxiety-like behaviors, we transiently activated PN projections to the BLA (PL^PNs^–BLA) through ChR2 optogenetic approach (Supplementary Fig. 6a, top). After AAV-DIO-ChR2-EGFP or AAV-DIO-EGFP was injected into the PL of CaMKIIα-Cre mice, optic fibers were implanted above the BLA of the mice (Supplementary Fig. 6a, bottom). In the OFT, selective optogenetic activation of the PL–BLA glutamatergic neuron terminals in BLA resulted in mice spending significantly less time on center exploration (Supplementary Fig. 6b). Meanwhile, in the EPM test, PL–BLA glutamatergic neuron terminals activation led to a significant reduction in the time spent on the open arms (Supplementary Fig. 6c).

To further explore whether PL–BLA circuit activation had an effect on autism-like behaviors, we conducted three-chamber test and grooming test. During three-chamber test, the mice showed no changes in social time with S1 in phase2 and with S2 in phase3 during PL–BLA circuit terminals optogenetic activations or not (Supplementary Fig. 6d, e). Moreover, no changes were observed in the grooming time with or without PL–BLA circuit activation (Supplementary Fig. 6f).

To further determine whether the PL–BLA circuit is selective for anxiety-like behaviors, we suppressed PL^PNs^–BLA via NpHR optogenetic approach to examine whether the behavioral abnormalities could be rescued in *Tmem74*^−/−^ mice (Supplementary Fig. 7a). In OFT and EPM test, selective optogenetic suppression PL–BLA circuit resulted in a significant increase in time exploring the center zone or the open arms (Supplementary Fig. 7b, c), indicating an obvious alleviation of anxiety phenotypes. Additionally, we found selectively suppressed PL–BLA circuit had no effects on the social novelty (Supplementary Fig. 7d, e) and grooming behavior (Supplementary Fig. 7f).

Together, these data suggested that hyperactivity of glutamatergic *Tmem74* deficit neurons in PL promote anxiety-like behaviors via the PL–BLA circuit.

### Anatomically distinct subpopulations of PL pyramidal neurons project to the STR and BLA

**Fig. 5:**
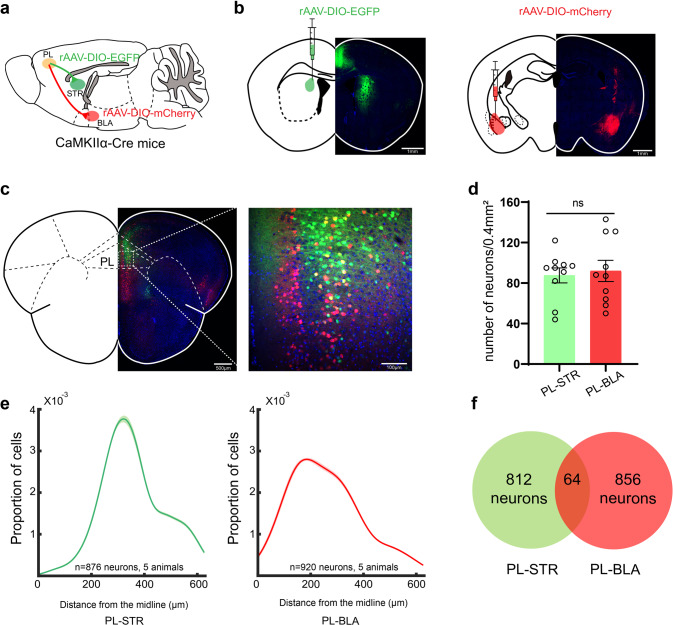
Anatomically distinct subpopulations of PL pyramidal neurons project to the STR and BLA. **a**, **b** Schematic and histology of AAV2-Retro-EF1α-DIO-EGFP-WPRE-hGHpA (rAAV-DIO-EGFP, green) injection into dSTR and AAV2-Retro-EF1a-DIO-mCherry-WPRE-hGHpA (rAAV-DIO-mCherry, red) injection into BLA in CaMKIIα-Cre mice. **c** A coronal brain slice (2.2 mm anterior bregma) of PL neurons in CaMKIIα-Cre mice after rAAV-DIO-EGFP injection into dSTR and rAAV-DIO-mCherry injection into BLA. **d** Quantification of number of PL pyramidal neurons labeled with green from dSTR injections and red from BLA injections (*n* = 5 in each group, and two views of slices were provided for each mouse). **e** Quantification of the medial/lateral pyramidal neurons distribution of PL–STR (green) and PL–BLA (Red). Shading denotes SEM (The two groups are distributed differently, *p* = 0.0118). **f** An overlapping Venn diagram of the PL–dSTR neurons and PL–BLA neurons (neuron overlap:3.56%). Data were presented as means ± SEM. *P < 0.05; ns not significant. Unpaired two-tailed Student’s *t*-test for **d**; Kolmogorov-Smirnov test for **e**.

To further identify the neural circuits of PL^PNs^–dSTR^MSNs^ and PL^PNs^–BLA^PNs^, retrograde tracer, AAV2-Retro-EF1α-DIO-EGFP-WPRE-hGHpA (rAAV-DIO-EGFP) or AAV2-Retro-EF1a-DIO-mCherry-WPRE-hGHpA (rAAV-DIO-mCherry), was injected respectively into the dSTR or BLA of CaMKII-Cre mice to track each target region (Fig. 5a, b). mCherry-positive and EGFP-positive neurons appeared in the PL of CaMKII-Cre mice, and mCherry-positive PNs were positioned more medially than EGFP-positive PL–dSTR PN neurons (Fig. 5c, e, and Supplementary Fig. 8a, b). Similar densities of mCherry-positive and EGFP-positive neurons were observed in the PL (Fig. 5d). Interestingly, there was little overlap between the two subpopulations (Fig. 5f). To further compare PL–dSTR and PL–BLA circuit neuron electrophysiological characteristics, we performed whole-cell patch clamp recordings of the two subpopulations. Our data showed that there were no significant differences between the two subpopulations in terms of membrane properties, AP firing rates, or AP parameters (Supplementary Fig. 8c–j).

### Enrichment of TMEM74 in PL pyramidal neurons alleviated the comorbidity of autism- and anxiety-like behaviors

**Fig. 6:**
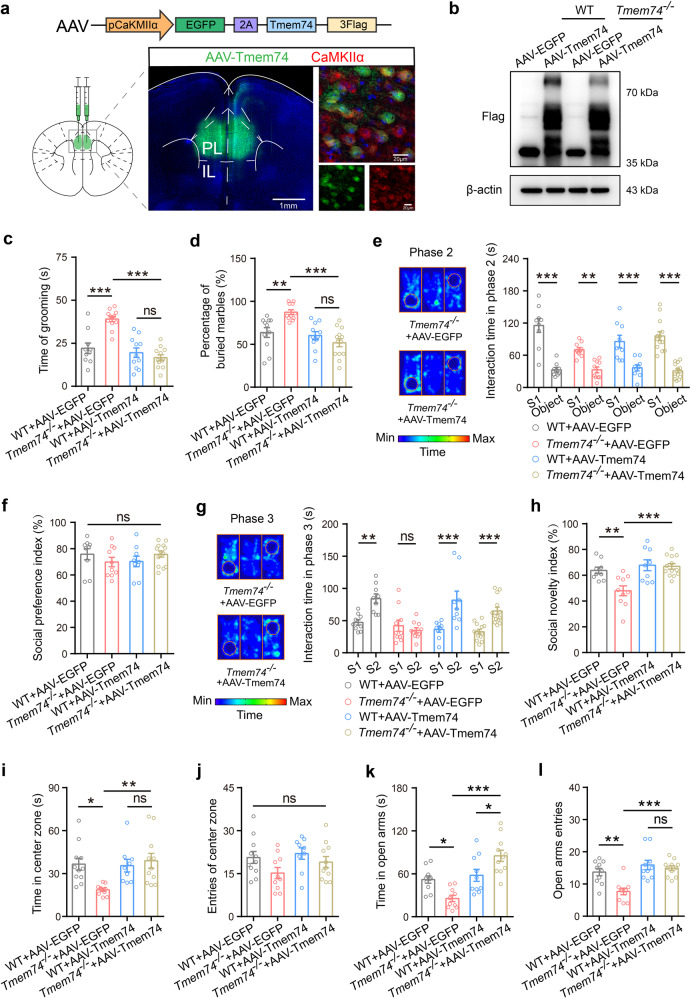
Overexpression of TMEM74 in PL rescues social deficits and anxiety-like behaviors. **a** Schematic of pAAV2/9-CaMKIIα-EGFP-2A-Tmem74-3FLAG (AAV-Tmem74) constructs and representative images of EGFP (green) and CaMKIIα (red) after AAV-Tmem74 injection to PL. **b** Representative immunoblotting band of Flag from PL after AAV-EGFP or AAV-TMEM74 injection to PL in WT and *Tmem74*^−/−^ mice. **c**–**h** Quantification of the grooming time (**c**, *n* = 10 WT + AAV-EGFP mice, *n* = 12 *Tmem74*^−/−^+AAV-EGFP mice, *n* = 11 WT + AAV-Tmem74 mice, *n* = 12 *Tmem74*^−/−^+AAV-Tmem74 mice), the percentage of buried marble (**d**, *n* = 11 WT + AAV-EGFP mice, *n* = 11 *Tmem74*^−/−^+AAV-EGFP mice, *n* = 11 WT + AAV-Tmem74 mice, *n* = 13 *Tmem74*^−/−^+AAV-Tmem74 mice), the interaction time (**e**) and the sociability preference index (**f**) with S1 mouse in the phase 2, and the time spent interacting (**g**) and social novelty index (**h**) with novel S2 in the phase 3 of three-chamber tests (*n* = 9 WT + AAV-EGFP mice, *n* = 10 *Tmem74*^−/−^+AAV-EGFP mice, *n* = 9 WT + AAV-Tmem74 mice, *n* = 13 *Tmem74*^−/−^+AAV-Tmem74 mice) after AAV-EGFP or AAV-TMEM74 injection to PL. **i**, **j** Quantification of the time in central zone (**i**) and the entries of central zone (**j**) (*n* = 11 WT + AAV-EGFP mice, *n* = 10 *Tmem74*^−/−^+AAV-EGFP mice, *n* = 9 WT + AAV-Tmem74 mice, *n* = 11 *Tmem74*^*-/-*^+AAV-Tmem74 mice) in the open field test. **k**, **l** Quantification of the time spent in open arms (**k**) and number of entries to open arms (**l**) (*n* = 10 WT + AAV-EGFP mice, *n* = 10 *Tmem74*^−/−^+AAV-EGFP mice, *n* = 10 WT + AAV-Tmem74 mice, *n* = 11 *Tmem74*^−/−^+AAV-Tmem74 mice) in the elevated plus maze. Data were presented as means ± SEM. *P < 0.05, **P < 0.01, ***P < 0.001; ns not significant. One-way ANOVA followed by Tukeyʼs post hoc test for **c**, **d**, **h** and **i–l**; One-way ANOVA followed by Bonferroni’s post hoc test for **f**; Two-way ANOVA followed by Bonferroni’s post hoc test for **e**; Two-way ANOVA followed by Sidak’s post hoc test for **g**.

To further confirm the hypothesis that a *Tmem74* deficit in the PL mediated social deficits and anxiety-like behaviors, pAAV2/9-CaMKIIα-EGFP-Tmem74-3Flag or pAAV2/9-CaMKIIα-EGFP-3Flag was injected respectively into the PLs of 4-week-old WT and *Tmem74*^−/−^ mice (Fig. 6a). The expression of 3-Flag was used to identify the expression of Tmem74 in the PL (Fig. 6b). Through whole-cell patch clamp recordings, we found that introducing Tmem74 in PNs of *Tmem74*^−/−^ mice could rescued the abnormal electrophysiological characteristics such as Tau, Rin and AP frequency to the level of wild-type PNs (Supplementary Fig. 9b–d), but no effects on RMP and other AP parameters (Supplementary Fig. 9a, e–h). Then, we performed autism- and anxiety-like behavior analyses. In autism-like behavior analyses, *Tmem74*^−/−^ mice displayed increased repeated grooming phenotypes and numbers of marbles buried, both of which were alleviated by a gain of TMEM74 (Fig. 6c, d). In the three-chamber tests, all mice in the four groups spent more time exploring the S1 mouse than the object on the other side, and the social preference index was not altered in the phase 2 (Fig. 6e, f). However, the differences between WT and *Tmem74*^*-/-*^ mice were attenuated by an enrichment of Tmem74 in the PL during the phase 3 (Fig. 6g, h). WT mice injected with AAV-Tmem74 had normal behavioral phenotypes compared with the WT-EGFP group. Taken together, these data indicated the regulatory role of *Tmem74* in the PL for stereotyped behavior and social novelty.

To determine whether anxiety-like behaviors could be reversed by an enrichment of Tmem74 in *Tmem74*^−/−^ mice, we performed OFT and EPM analyses. In the OFT, *Tmem74*^−/−^ mice spent less time on moving in the central zone and more time in the surrounding zone, which was augmented in the *Tmem74*^−/−^+AAV-Tmem74 group (Fig. 6i and Supplementary Fig. 9i). The numbers of entries into the center zone and the total distance in the open field were not changed in any of the groups (Fig. 6j and Supplementary Fig. 9j). In the EPM test, *Tmem74*^−/−^ mice spent less time on the open arms, more time on the closed arms, and had fewer numbers of entries into the open arms than WT mice, which were improved in the *Tmem74*^−/−^+AAV-Tmem74 group (Fig. 6k, l, and Supplementary Fig. 9k, l).

## Discussion

Here, we identify that divergent PL projections mediate autism- and anxiety-like behaviors. Notably, we reveal that the anatomy and function of PL projections to the dSTR and BLA play a role in stereotyped, social, and anxious behaviors. Namely, *Tmem74* deficits activate the two assemblies of pyramidal neurons in the PL, which projects to dSTR and BLA respectively. The activation of PL–dSTR circuit neurons contributes to autism-like behaviors, whereas the activation of PL–BLA circuit neurons leads to anxiety-like behaviors. Meanwhile, suppression of the two distinct circuit could rescue ASD-like and anxiety behaviors selectively. In summary, these findings further demonstrate that PL pyramidal neurons with distinct spatial positions regulate specific behaviors, which expands our understanding of the pathogenic mechanism of autism and anxiety comorbidity.

*Shank3b*^−/−^ mice and *Mir137* and *Shank2* mutant mice were reported as ASD models with deficits in social interaction [30, 43, 44]. Here, transcriptomics profiling analysis revealed the downregulation of *Tmem74* mRNA expression in three kinds of ASD mice. In addition, *Tmem74*^−/−^ mice expressed repetitive behavior, social novelty impairment, and autism-like behaviors with high c-Fos expression in the PNs of the PL, and decreased TMEM74 protein levels appeared in the PLs of *Shank3b*^−/−^ mice. Moreover, our previous studies have suggested that *Tmem74* deficits induce anxiety-like behavior [29]. In this context, we hypothesize that there is a relationship between PL *Tmem74* deficits and autistic- and anxiety-like behaviors. To verify this hypothesis, we employed *sgRNA* to specifically knock down *TMEM74* in the PNs of the PL of Cas9 mice through a CRISPR/Cas9 system [29]. Consistent with this hypothesis, we found that PL pyramidal neuronal *TMEM74*-deficient mice showed a hyperactivity of PNs, stereotyped locomotion, social deficiencies, and anxious behaviors. Our findings provide a novel rodent model of autism and anxiety comorbidity as well as new insights into the mechanisms of the pathogenesis of ASD and anxiety triggered by *Tmem74* deficiency.

The active circuits of PL projections, such as those of the PL–NAc circuit, cause a change in social preferences [16], while PL–VTA or PL–PVT circuit activation is necessary for the retrieval of morphine withdrawal memory [45] and the locomotion velocity in the 3-Chamber Assay [16], respectively. Here, our study sheds light on a previously unrecognized role of activation of distinct PL–dSTR/BLA pathways differentially drives autism- and anxiety-like behaviors. Notably, glutamatergic projections from the PL to distinct brain areas play an important role in emotion. The glutamatergic PL–NAc circuit was reported to be involved in anxiety- and obsessive-compulsive-like behaviors [46]. The PL pyramidal neurons projecting to the BLA are important in the control of cognition and emotion [47]. Through gene regulation in combination with ex vivo and in vivo neural recordings in mice, our findings demonstrate that active glutamatergic projections from the PL to the BLA mediate pyramidal neuron hyperactivity and anxiety-like behaviors and that to the dSTR mediates FSI hyperexcitability and MSN inhibition and autism-like behaviors. More significantly, the two populations of PL projecting PNs belong to different spatial locations, the PNs projecting to the BLA are positioned more medially than those projecting to the STR. Given the heterogeneity of PL subregions and the different functions of the PL subregions in behavior, a previously unrecognized neuronal circuitry for autism-anxiety comorbidity is revealed.

In conclusion, we found that abnormalities in the PL–dSTR circuit lead to autistic behavior, and abnormalities in the PL–BLA circuit lead to anxious behavior. Further understanding of the regulation of these circuits might provide specific therapeutic opportunities for ameliorating autism and anxiety comorbidity. The restoration TMEM74 function reversed autism-related impairments and anxiety-like behavior, highlighting the central role of TMEM74 signal in the regulation of emotional state. Thus, these studies support that the neural circuits of the PL are functionally diverse. Moreover, the present findings increase the mechanistic understanding of the prelimbic cortex-regulated circuits involved in autism- and anxiety-like behavior, which might lead to the development of new therapeutic avenues to improve the well-being of patients.

### Supplementary information

Supplementary methods and materials

## Data Availability

pCLAMP 10.3 software (Molecular Devices, Sunnyvale, CA, USA) and Matlab software (MathWorks, Natick, MA, USA) were used for electrophysiology data analysis. All codes are available from the authors upon reasonable request.
