# Supplementary material for: Divergent projections of the prelimbic cortex mediate autism- and anxiety-like behaviors
Source: Mol Psychiatry. 2023 Jan 23;28(6):2343–54. doi: 10.1038/s41380-023-01954-y (PMC10611563; doi:10.1038/s41380-023-01954-y)
Supplement: Supplementary file 1 — Supplementary methods and materials [file 41380_2023_1954_MOESM1_ESM.docx]

**Supplementary materials for**

**Divergent projections of the prelimbic cortex mediate autism- and anxiety-like behaviors**

Yi-Fan Luo^1,5^, Lu Lu^1,5^, Heng-Yi Song^1^, Han Xu^1^, Zhi-Wei Zheng^2^, Zhou-Yue Wu^1^, Chen-Chen Jiang^1^, Chu Tong^2^, Hao-Yang Yuan^2^, Xiu-Xiu Liu^1^, Xiang Chen^1^, Mei-ling Sun^1^, Ya-Min Tang^1^, Heng-Yu Fan^3^, Feng Han^1,4*^, Ying-Mei Lu^2,4*^

^1^International Joint Laboratory for Drug Target of Critical Illnesses, Key Laboratory of Cardiovascular and Cerebrovascular Medicine, School of Pharmacy, Nanjing Medical University, Nanjing 211166, China. ^2^Department of Physiology, School of Basic Medical Sciences, Nanjing Medical University, Nanjing 211166, China. ^3^Life Sciences Institute and Innovation Center for Cell Biology, Zhejiang University, Hangzhou, China. ^4^Institute of Brain Science, the Affiliated Brain Hospital of Nanjing Medical University, Nanjing 211166, China.

^5^These authors contributed equally.

Running head: PL projections mediate autism comorbidity

^*^Email: fenghan169@njmu.edu.cn, lufx@njmu.edu.cn

This file includes:

Supplementary Materials and Methods

Supplementary Figure 1 to 9

**Supplementary methods and materials**

**Animals.** *Tmem74^-/-^* mice were generated as previously described [1] by knocking out the 8th base-an adenine after the promoter of the second exon of *Tmem74* gene, and the subsequent base sequence had a frameshift mutation. All *Tmem74^-/-^* mice, *C57BL/6J* strain (SHANGHAI SLAC, Shanghai, China) mice, Rosa26-LSL-Cas9 knock-in mice (Stock No: 024857, Jackson Laboratory), *Shank3* knockout mice (a gift from Wei Lu, Southeast University) [2, 3] and *B6.Cg-Tg(CaMKIIα-Cre)T29-1Stl/J* (*CaMKIIα-Cre*) (stock no. 007914, Jackson Laboratory) mice were housed at 22-23℃ and on a 12-h light/12-h dark cycle with *ad libitum* access to water and food. Animal procedures were approved by the Animal Advisory Committees at Nanjing Medical University for the Care and Use of Laboratory Animals and conformed to National Institutes of Health Guide for the Care and Use of Animals in China.

**Virus vectors.** For the chemogenetic manipulation, pAAV2/9-CaMKIIα-hM4D(Gi)-mCherry-3FLAG (AAV-CaMKIIα-hM4D(Gi)-mCherry, 5.89 × 10^12^ particles/ml) was purchased from Obio Technology (Shanghai, China). For selective knockout of *Tmem74*, we designed the sgRNA sequences and genomic primers as previously described [1](sgRNA1: 5’-AGCTCCACTCTCTGTCTAAG-3’; sgRNA2: 5’-AGTGGACCCATGCAATGCTT-3’; sgRNA3: 5’-CTCTCTGTCTAAGAGGAACA-3’) according to CRISPR tool (http://crispr.mit.edu). The most efficient sgRNA1 was used in the subsequent *in vivo* *Tmem74* target. pAAV2/9-CaMKIIα-EGFP-2A-MSC-3FLAG (AAV-EGFP, 1.25 × 10^13^ particles/ml) or pAAV2/9-U6-sg*Tmem74*-CaMKIIα-Cre-HA-WPRE-hGHpA vector (AAV-sg*Tmem74*, 2 × 10^12^ particles/ml) (Obio Technology, Shanghai, China) was injected into Cas9 mice. For the optogenetic tests, pAAV2/9-EF1α-DIO-EGFP-WPRE (AAV-DIO-EGFP, 5.81 × 10^13^ particles/ml) or pAAV2/9-EF1α-DIO-hChR2 (H134R)-EGFP-WPRE (AAV-DIO-ChR2-EGFP, 1.27 × 10^13^ particles/ml) was from Obio Technology (Shanghai, China), rAAV2/9-CaMKIIα- mCherry-WPRE (AAV-CaMKIIα-mCherry, 5.73 × 10^12^ particles/ml) or rAAV2/9-CaMKIIα-eNpHR3.0-mCherry-WPRE (AAV- CaMKIIα-NpHR-mCherry, 5.40 × 10^12^ particles/ml) was from Brain VTA Co., Ltd (Wuhan, China). For the retrograde tracing, AAV2-Retro-EF1α-DIO-EGFP-WPRE-hGHpA (rAAV-DIO-EGFP, 5.22 × 10^12^ particles/ml) and AAV2-Retro-EF1α-DIO-mCherry-WPRE-hGHpA (rAAV-DIO-mCherry, 5.43 × 10^12^ particles/ml) were from Brain VTA Co., Ltd (Wuhan, China). For the overexpression of *Tmem74*, pAAV2/9-CaMKIIα-EGFP-2A-MCS-3FLAG (AAV-EGFP) or pAAV2/9-CaMKIIα-EGFP-2A-Tmem74-3FLAG vector (AAV-Tmem74) (Obio Technology, Shanghai, China) was injected into *Tmem74^-/-^* mice.

**Stereotaxic injection.** All viral injections were performed as previously [4]. In brief, mice were anesthetized with isoflurane gas/oxygen mixture (2%). The AAVs were microinfused with a glass pipette at 200 nl per side and 50 nl min^-1^ through a stereotaxic device (RWD Life Science) at the following stereotaxic coordinates: PL (AP: +1.90 mm，ML: ±0.40 mm; DV: -1.80 mm), dSTR (AP: +0.50 mm; ML: ±1.40 mm; DV: -3.00 mm) and BLA (AP: -1.22 mm; ML: ±3.25 mm; DV: -4.90 mm). Relative to bregma, AP, ML and DV denote anteroposterior, mediolateral and dorsoventral, respectively. For retrograde tracing of dSTR-projecting and BLA-projecting PL pyramidal neurons, retrograde tracing virus (200 nl) was injected respectively into the dSTR and BLA. Mice with virus mis-injections were excluded for analysis.

**Optical fiber and electrode implantation.** A ceramic ferrule with optical fiber (200μm in diameter, NA 0.37) was bilaterally implanted with the fiber tip into the dSTR (AP: +0.50 mm; ML: ±1.40 mm; DV: -3.00 mm) and above BLA (AP: -1.22 mm; ML: ±3.20 mm; DV: -4.80 mm) after the AAV injection. After implantation for 3 week, optogenetic activation experiments were conducted through a 472 nm blue laser diode, and the output of the laser at the tip of the fiber was measured using a Master-9 pulse stimulator (A.M.P.I.) and adjusted to 5.45mW (20-Hz trains with 5-ms pulses) before the behavioral analysis. Optogenetic suppression experiments were conducted through a 589 nm yellow laser diode, and the output of the laser at the tip of the fiber was measured using a Master-9 pulse stimulator (A.M.P.I.) and adjusted to 10mW. Mice with incorrect fiber implantation were excluded for analysis.

To monitor neuron activity, we implanted the tetrodes into the dSTR unilaterally (AP: +0.50 mm; ML: +1.40 mm; DV: -3.00 mm). Each tetrode consists of eight polyimide-coated nichrome wires connected to a 32-channel electrode interface board (EIB-32, Neuralynx). After implantation for 3 weeks, neurophysiological signals under behavioral tests were digitized using the Neuralynx Digital Lynx system via a multiplexing digital headstage. Spike channels were acquired at 40 kHz with 16-bit resolution, and band-pass filtered at 150Hz-3kHz before spike sorting. For spike sorting, a single unit was discriminated using an offline sorter (Plexon), and neurons were separated into FSIs and MSNs on the firing rate, waveform and waveform energy features. All data analyses were performed using MATLAB 2014b (The Mathworks, Inc., Natick, MA, USA). Mice with incorrect electrode implantation were excluded for analysis.

**Behavioral tests.** All mice (aged 8-12 weeks) were transported to the behavior test room. After one week of adaptation, the mice were performed for autism- and anxiety-like behaviors and memory-related behaviors. Mice were assigned to experimental groups based on their genotype (no randomization) or randomly allocated to groups. All experimental areas were cleaned with 75% ethanol before the tests. Data were collected by the software (ANY-maze, Stoelting, USA).

To analyze the stereotyped behaviors of mice, we perform the grooming behavior test and marble burying test. For the grooming behavior test, mice were placed in the open field box freely and allowed to roam freely for 10 min. The grooming time within 10 min was counted by randomized double-blind method [5]. In optogenetic manipulation grooming test, a blue light or yellow light was turned on during the 10 min test period. For Marble burying test, mice were subjected in a new 40×40×40 cm square box with 5-cm deep wood chips covering the bottom. A total of 36 colorful glass marbles were placed equidistantly in a 6×6 arrangement. Test was recorded for 30 min and unburied marbles were counted after the test. The marbles at least half buried in the sawdust were counted. The percentage of the buried marbles is calculated as the number of beads buried divided by total number of beads.

To analyze the social behaviors of mice, we conduct the three-chamber social test and home-cage test. Three-chamber social test. For the three-chamber social test, the three-chamber apparatus was 60×40×40 cm with 20-cm-wide middle and side chambers. In the phase 1, mice were put into the center chamber to explore the environment freely for 10 min. To test social preference (phase 2), strange mouse 1 (S1) was put in one of the cages when the other cage was empty. The test mouse was guided to the center chamber, then open gates to the side chambers. The test mouse was allowed to explore three chambers freely for 10 min. To test social novelty preference (phase 3), strange mouse 2 (S2) was placed in the empty cage and the test mouse was allowed to freely explore chambers again for 10 min. All the strange mice were male and randomly selected each time. The time of sniffing the strange mouse and the empty object (O) were recorded. Social preference index and social novelty index were calculated as the time spent exploring the targets (S1 or S2) divided by the total time spent exploring both targets (S1 and O, or S2 and S1) [6, 7]. In optogenetic manipulation three-chamber social test, a blue light or yellow light was turned on for 10 min in each phase. Each phase lasts for 10 min. For the home-cage test, mice were habituated in the home-cage for 1 min before an unfamiliar juvenile mouse was guided into the cage for a 5 min period. Body sniffing, anogenital sniffing, direct contact and close following(＜1 cm) initiated by the test mice were defined as social investigation.

To assess the instinctive behavior of mice, nesting test is performed. Mice were individually placed in the home cage and adapted for 24 h. A 5×5 cm piece of cotton weighing about 2 g was placed in the same place of each cage. Photographs were taken 48 h later to assess the nesting condition. The scoring criteria were as follows [8]: 0-the cotton was unbitten, 1-a little of the cotton was bitten and no obvious nesting site, 2-most of the cotton was bitten into strip, 3-the cotton was gathered into a clear nesting site, 4-a complete nesting shape was formed and higher than the body of the mouse.

To examine the anxiety-like behavior of mice, open field test and elevated plus maze test are performed [9]. For the open field test, mice were placed into the open field box (50×50×45 cm) with a 20-cm-wide square central zone, and their movements were recorded with video cameras for 5 min. Anxiety-like behavior was defined by the time and entries in the central zone. Locomotor activity was calculated as total traveled distance. In optogenetic manipulation open field test, a blue light or yellow light was turned on during the 5 min test period. For the elevated plus maze test, the apparatus comprised two open arms (30×5 cm) and two closed arms (30×5×14 cm) elevated about 60 cm above the surface. Mice were introduced to the central zone with the same head orientation to freely explore for 8 min. Anxiety-like behaviors were calculated by the time and number of entries in the open arms or closed arms. In optogenetic manipulation elevated plus maze test, a blue light or yellow light was turned on for 8 min and each test lasts for 8 min.

To check memory and recognition of mice, Y-maze test and novelty object recognition test were performed [10, 11]. For the Y-maze test, mice were placed at the end of one same arm, with the head facing the wall. Mice could move freely for 8 min in three closed arms and the tracks were recorded by the camera. Three consecutive choices of all three arms were considered as a correct alternation. The percentage of spontaneous alternations was calculated as: [the number of correct alternations / (the total number of choices -2)] ×100% [10]. For novelty object recognition test, during training, two identical cuboid objects were placed in the 50×50×45 cm box and mice explored them for 5 min. 4 h later, one of the training objects was replaced by a novel triangle object in the 5-min test session. The time of sniffing familiar and novel objects were compared to examine whether mice had an innate preference for novelty. Discrimination index was defined as: (the sniffing time of the novel object) / [(the sniffing time of the familiar object + the sniffing time of the novel object)] × 100% [11].

**Brain slice preparation.** Postnatal 21- to 28-day-old male mice were anesthetized and then decapitated. As described previously [4], brains were quickly removed to ice-cold oxygenated cutting solution. The cutting solution contained the following (in mmol/L): 75 sucrose, 87 NaCl, 2.5 KCl, 1.25 NaH_2_PO_4_, 25 NaHCO_3_, 0.5 CaCl_2_, 7 MgCl_2_ and 25 glucose. Slices (300 μm thick) were cut by the vibratome (VT1000S Leica) and then transferred to normal artificial cerebrospinal fluid (ACSF) containing (in mmol/L): 124 NaCl, 3 KCl, 1.25 NaH_2_PO_4_, 26 NaHCO_3_, 2 CaCl_2_, 1 MgSO_4_ and 10 glucose. The slices were incubated in ACSF for 30 min at 34℃ and then maintained at room temperature (24 ± 1℃) for 1 h. All external solutions were saturated with 95% O_2_/5% CO_2_. Mice with AAV injection were used at 3 weeks.

**Whole-cell recordings.** Pyramidal neurons of PL and BLA were recorded with a MultiClamp 700B amplifier and 1550A digitizer (Molecular Devices). Neuronal cells with EGFP were visualized with a laser optics microscope equipped with 40× lens (Olympus). For slices, the recording electrode resistance ranged from 3.5-5.5 MΩ. Neurons were held at -70 mV, with the pipette solution containing (in mmol/L): 130 K-gluconate, 20 KCl, 10 HEPES, 0.2 EGTA, 4 Mg-ATP and 0.5 Na_3_-GTP (pH was adjusted to 7.30 with KOH). The membrane time constant (Tau) was fit by an exponential function of the membrane potential change in response to a rectangular hyperpolarizing current stimulation and induced a small (about 3-5 mV) voltage deflection. Input resistance (Rin) was induced by a gradient hyperpolarizing current of -60-10 pA stepped 10 pA and was calculated from the slope in the linear phase of current-voltage plots. For the action potential (AP) recording, we used 0-300 pA depolarizing current stepped 20 pA. We analyzed the first spike evoked by the minimum current to acquire AP properties. Membrane properties and AP firing were measured by current-clamp recording. Data were analyzed using Clampfit 10 (Molecular Devices) and MATLAB (MathWorks). All drugs and regents were from Sigma or Tocris.

**Drug treatment.** For the behavior test with DREADD-hM3Di, CNO (Sigma Aldrich, Cat# C0832) was dissolved in DMSO and diluted with saline, the final concentration of DMSO was 0.5%. Mice were intraperitoneally injected with saline or CNO (1 mg/kg). In the electrophysiological recording, the CNO final concentration was 10 μmol/L.

**Western blotting.** Brain samples were dissected, and homogenized in lysis buffer as described previously [12]. In brief, primary antibodies were used as shown: TMEM74 (1:1000; Sigma Aldrich, Cat# SAB2103292), Flag (1:2000; Proteintech, Cat# 20543-1-AP), β-actin (1:5000; Multi Sciences, Cat# ab-008). An enhanced Chemiluminescence Detection Kit (Biological Industries) was used for visualization of immunoreactive proteins. Intensity of bands was quantitated by using ImageJ (USA, NIH) and the results were normalized to the loading control (β-actin).

**Immunohistochemistry.** As previously described [13], the brain tissue was cut into 40-μm-thick slices by the freezing microtome (Leica CM 1950) and stored in the cryoprotectant containing: PBS 50%, glycol 30%, and glycerol 20%. The slices were washed with PBS for three times (10 min each time). After incubation in 0.1% Triton X-100 for 15 min and in BSA for 1 h at room temperature, the brain sections were incubated with the following primary antibodies for 48 h: TMEM74 (1:300, Sigma Aldrich, Cat# SAB2103292), CaMKIIα (1:300) [14], c-Fos (1:1000; Synaptic Systems, Cat# 226003), PV (1:5000; Swant, Cat# 235), DAPI (1:500, Thermo Fisher Scientific, Cat# D1306), and then exposed to fluorescent secondary antibodies for 4 h. After washed by PBS for three times, slices were visualized by confocal microscope (Zeiss LSM 800) and analyzed by ImageJ software (USA, NIH).

To ascertain retrograde labeling of PL-STR and PL-BLA neurons, coronal sections containing PL were imaged (Zeiss LSM 800). Three PL sections of each mouse were chosen along the anterior-posterior axis (~1.8 mm, ~2.2 mm, and ~2.6 mm anterior to bregma) and two symmetrical views of each section were selected for each mouse. The distance from the midline of each cell and their M/L distributions were measured.

**Identification of differentially expressed (DE) genes.** Three ASD-related databases (GSE79824, GSE132684, GSE79661) were used to screen the intersection of DE genes with a *P*-value < 0.05. Five overlapping DE genes were obtained (*Alpk1*, *Dock9*, *Shank2*, *Syne1* and *Tmem74*), and *Tmem74* was further downregulated in the human pluripotent stem cells with ASD-related mutations in the GSE146760.

**Quantification and statistical analyses.** The sample size (n) for experiments were described in each figure legend. The sample size was based on our previous works and met the standards generally used in the field [1, 4], and no statistical methods were used to predetermine sample size. Investigators were blinded to the groups and samples during the experiments for where applicable. The variance between the groups is similar. ImageJ was used for quantifying c-Fos and co-localization of EGFP and neurons. Statistical analyses were performed using GraphPad Prism 6. Data analyzed by unpaired *t* test were pretested for equal variance by *F*-test. For two normally distributed groups, significances were calculated by unpaired two-tailed Student’s *t*-test. One-way ANOVA followed by Tukey’s post hoc test or two-way ANOVA followed by Sidak’s post hoc test were used for multiple-group comparisons. Kolmogorov-Smirnov test was used to compare M/L distribution of the 2 subpopulations of neurons in PL. All values represented using mean ± SEM. Significant differences are indicated when *P* value < 0.05 (**P* < 0.05, ***P* < 0.01, ****P* < 0.001).

**Reference:**

1. Shao LX, Jiang Q, Liu XX, Gong DM, Yin YY, Wu G *et al.* Functional coupling of Tmem74 and HCN1 channels regulates anxiety-like behavior in BLA neurons. Mol Psychiatry. 2019; 24: 1461-77.
2. Guo BL, Chen J, Chen Q, Ren KK, Feng DY, Mao HH *et al.* Anterior cingulate cortex dysfunction underlies social deficits in Shank3 mutant mice. Nat Neurosci. 2019; 22: 1223-34.
3. Peça J, Feliciano C, Ting JT, Wang WT, Wells MF, Venkatraman TN *et al.* Shank3 mutant mice display autistic-like behaviours and striatal dysfunction. Nature. 2011; 472: 437-42.
4. Tan C, Lu NN, Wang CK, Chen DY, Sun NH, Lyu H *et al.* Endothelium-Derived Semaphorin 3G Regulates Hippocampal Synaptic Structure and Plasticity via Neuropilin-2/PlexinA4. Neuron. 2019; 101: 920-37.
5. Folkes OM, Báldi R, Kondev V, Marcus DJ, Hartley ND, Turner BD *et al.* An endocannabinoid-regulated basolateral amygdala–nucleus accumbens circuit modulates sociability. J Clin Invest. 2020; 130: 1728-42.
6. Dong ZQ, Chen WB, Chen C, Wang HS, Cui WP, Tan ZB *et al.* CUL3 Deficiency Causes Social Deficits and Anxiety-like Behaviors by Impairing Excitation-Inhibition Balance through the Promotion of Cap-Dependent Translation. Neuron. 2020; 105: 475-90.
7. Nygaard KR, Maloney SE, Dougherty JD. Erroneous inference based on a lack of preference within one group: Autism, mice, and the social approach task. Autism Res. 2019; 12: 1171-83.
8. Dutta R, Lunzer MM, Auger JL, Akgün E, Portoghese PS, Binstadt BA. A bivalent compound targeting CCR5 and the mu opioid receptor treats inflammatory arthritis pain in mice without inducing pharmacologic tolerance. Arthritis Res Ther. 2018; 20.
9. Dimitrov EL, Tsuda MC, Cameron HA, Usdin TB. Anxiety- and Depression-Like Behavior and Impaired Neurogenesis Evoked by Peripheral Neuropathy Persist following Resolution of Prolonged Tactile Hypersensitivity. J Neurosci. 2014; 34: 12304-12.
10. Cao W, Lin S, Xia QQ, Du YL, Yang Q, Zhang MY *et al.* Gamma Oscillation Dysfunction in mPFC Leads to Social Deficits in Neuroligin 3 R451C Knockin Mice. Neuron. 2018; 97: 1253-60.
11. Lueptow LM. Novel Object Recognition Test for the Investigation of Learning and Memory in Mice. J Vis Exp. 2017;(126).
12. Wang H, Hong LJ, Huang JY, Jiang Q, Tao RR, Tan C *et al.* P2RX7 sensitizes Mac-1/ICAM-1-dependent leukocyte-endothelial adhesion and promotes neurovascular injury during septic encephalopathy. Cell Res. 2015; 25: 674-90.
13. Liu XX, Yang L, Shao LX, He Y, Wu G, Bao YH *et al.* Endothelial Cdk5 deficit leads to the development of spontaneous epilepsy through CXCL1/CXCR2-mediated reactive astrogliosis. J Exp Med. 2020; 217.
14. Fukunaga K, Goto S, Miyamoto E. Immunohistochemical localization of Ca2+/calmodulin-dependent protein kinase II in rat brain and various tissues. J Neurochem. 1988; 51: 1070-78.

**Supplementary Figures**


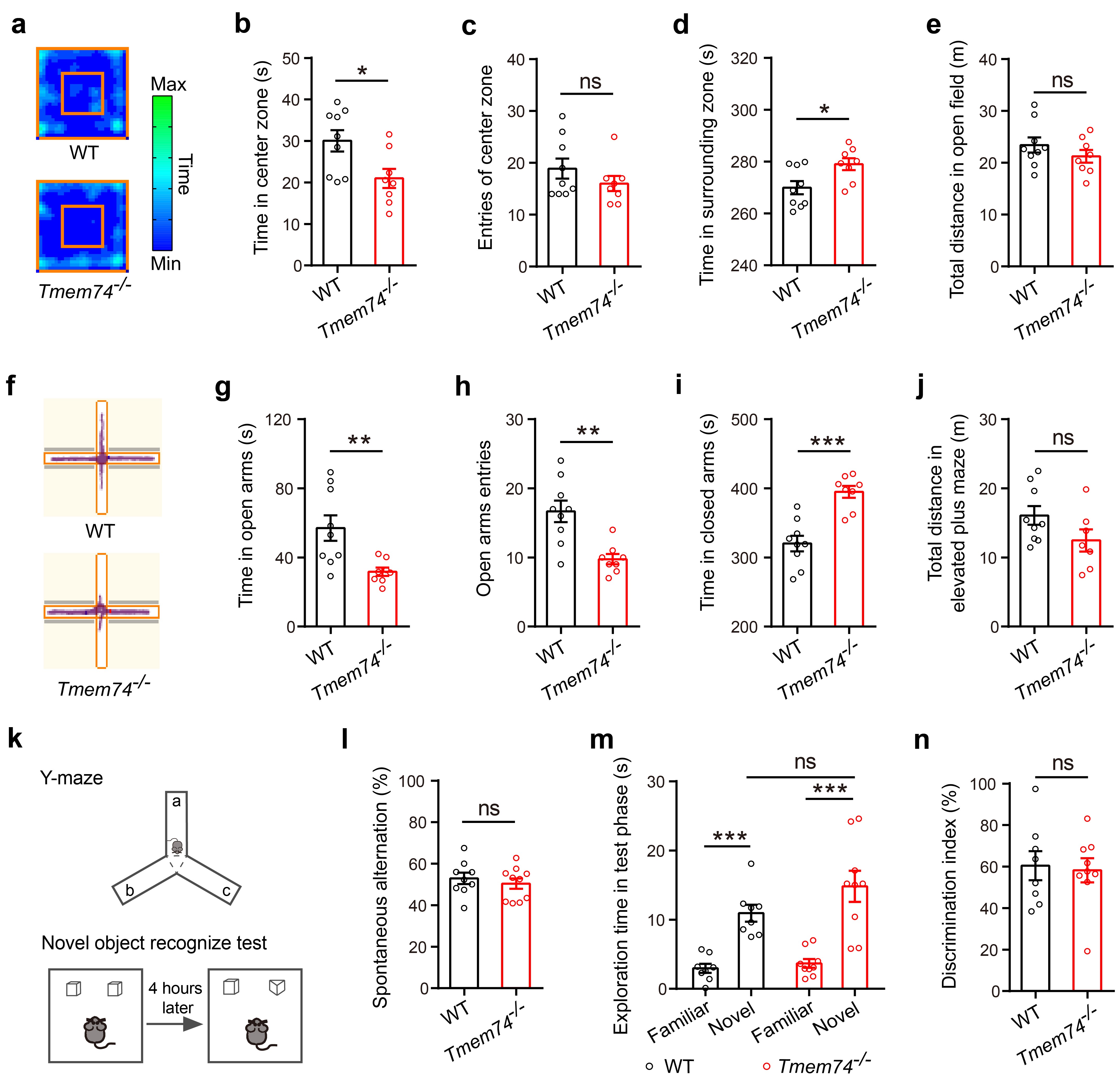


**Supplementary Figure 1 *Tmem74^-/-^* mice show anxiety-like behaviors.** (**a**) Representative heatmap in the open field test. (**b-e**) Quantitation of the time in center zone (**b**), entries of center zone (**c**), the time in surrounding zone (**d**) and total distance (**e**) in the 5-min open field test. (**f**) Representative traces of mice in the elevated plus maze test. (**g**-**j**) Quantitation of the time (**g)** and entries **(h)** in open arms, and the time in closed arms (**i**) and total moving distance (**j**) in WT and *Tmem74^-/-^* mice in the elevated plus maze test. (**k**) Behavior paradigms of Y-maze test and novelty recognition test. (**l**) Quantitation of spontaneous alternation in Y-maze test. (**m**, **n**) Quantitation of the time of exploring novel object (**m**) and the discrimination index (**n**) in the novel object recognition test. n=8 WT mice, n=9 *Tmem74^-/-^* mice. Data were presented as means ± SEM. **P* < 0.05, ***P* < 0.01, ****P* < 0.001; ns, not significant. Unpaired two-tailed Student’s t test for **b**-**e**, **g**-**j**, **l** and **n**; Two-way ANOVA followed by Sidakʼs post hoc test for **m**.


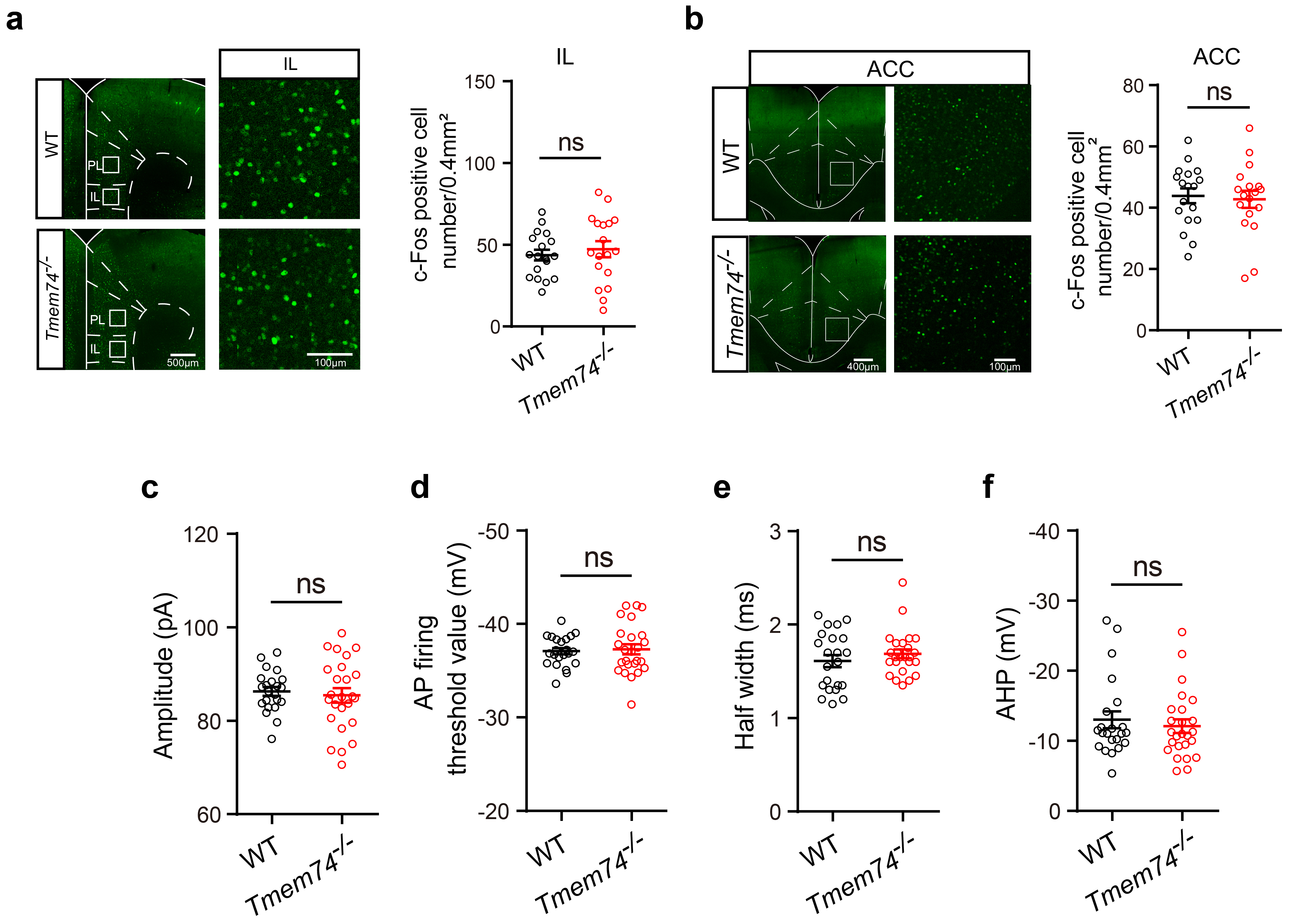


**Supplementary Figure 2 c-Fos expression after social interaction and AP properties in PL pyramidal neurons.** (**a**, **b**) Representative images and quantitation of c-Fos in IL (**a**) and ACC (**b**) after social interaction in WT and *Tmem74^-/-^* mice (n=3 in each group, and six views of slices from each mouse were provided). (**c**-**f**) Quantitation of the AP properties of PL pyramidal neurons (n=22 cells from 7 WT mice, n=25 cells from 5 *Tmem74^-/-^* mice). AP amplitude (**c**), AP firing threshold (**d**), AP half-width (**e**), AP AHP (**f**). Data were presented as means ± SEM. ns, not significant. Unpaired two-tailed Student’s t test was used.


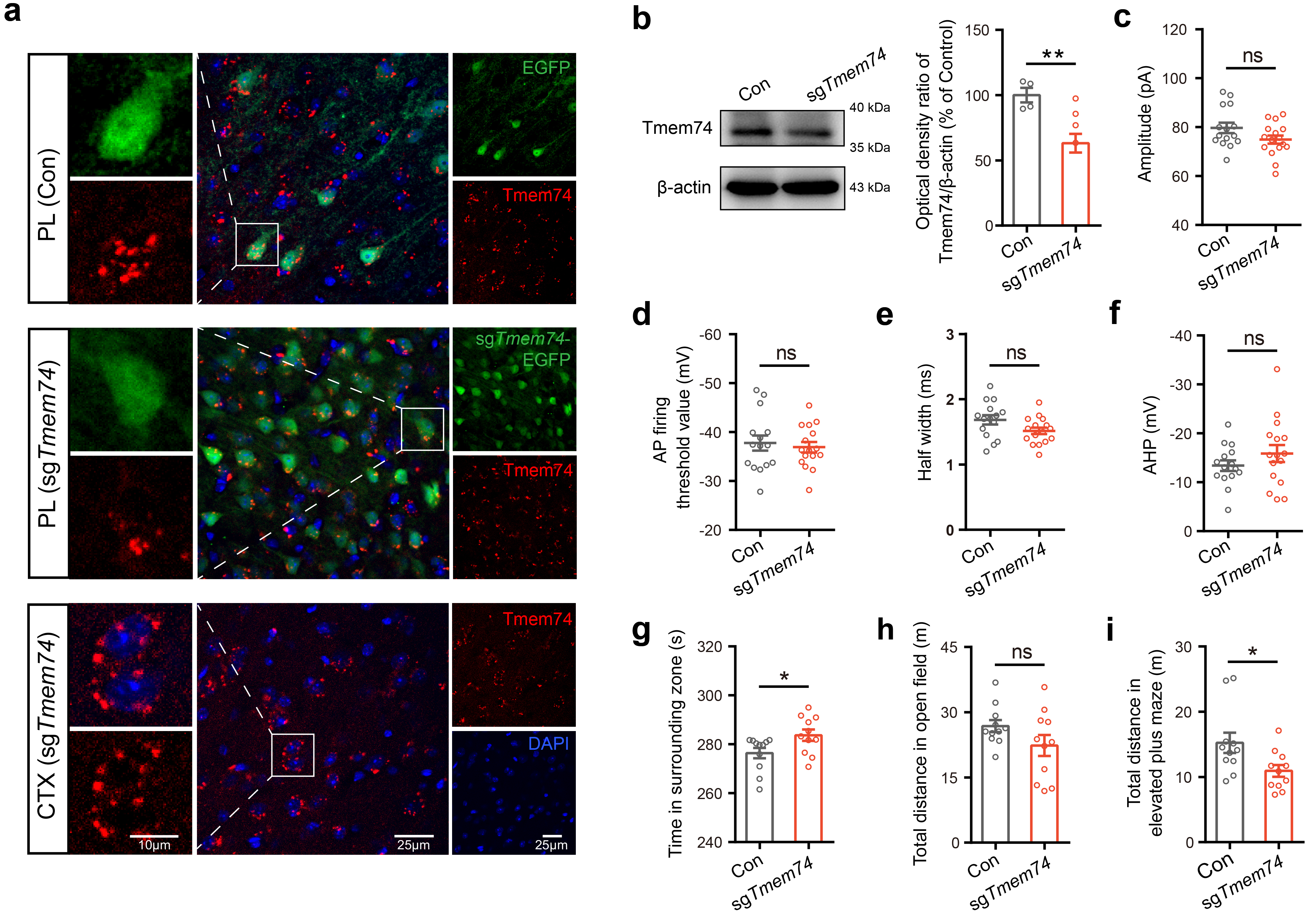


**Supplementary Figure 3 Effects of regional *Tmem74* deficit in pyramidal neurons on AP properties and anxiety-related behavior.** (**a**) Representative images showing the efficient knockout of sg*Tmem74* after AAV-EGFP (Con) or AAV-sg*Tmem74* (sg*Tmem74*) injection to Cas9 mice. CTX, cortex. (**b**) Representative band and quantitation of TMEM74 in AAV-EGFP injection mice (Con) or AAV-sg*Tmem74* injection mice (sg*Tmem74*) by western blot analysis (n=4 in each group). (**c-f**) Quantitation of AP properties of PL pyramidal neurons (n=15 cells from 4 control mice, n=16 cells from AAV-sg*Tmem74*-injected mice. AP amplitude (**c**), AP firing threshold (**d**), AP half-width (**e**) and AHP (**f**). (**g**) Quantitation of the exploring time in the surrounding zone. (**h**) Quantitation of total distance in the open field test in both groups. (**i)** Quantitation of the moving distance in the elevated plus maze. n=11 in each group. Data were presented as means ± SEM. **P* < 0.05, ***P* < 0.01; ns, not significant. Unpaired two-tailed Student’s t test was used.


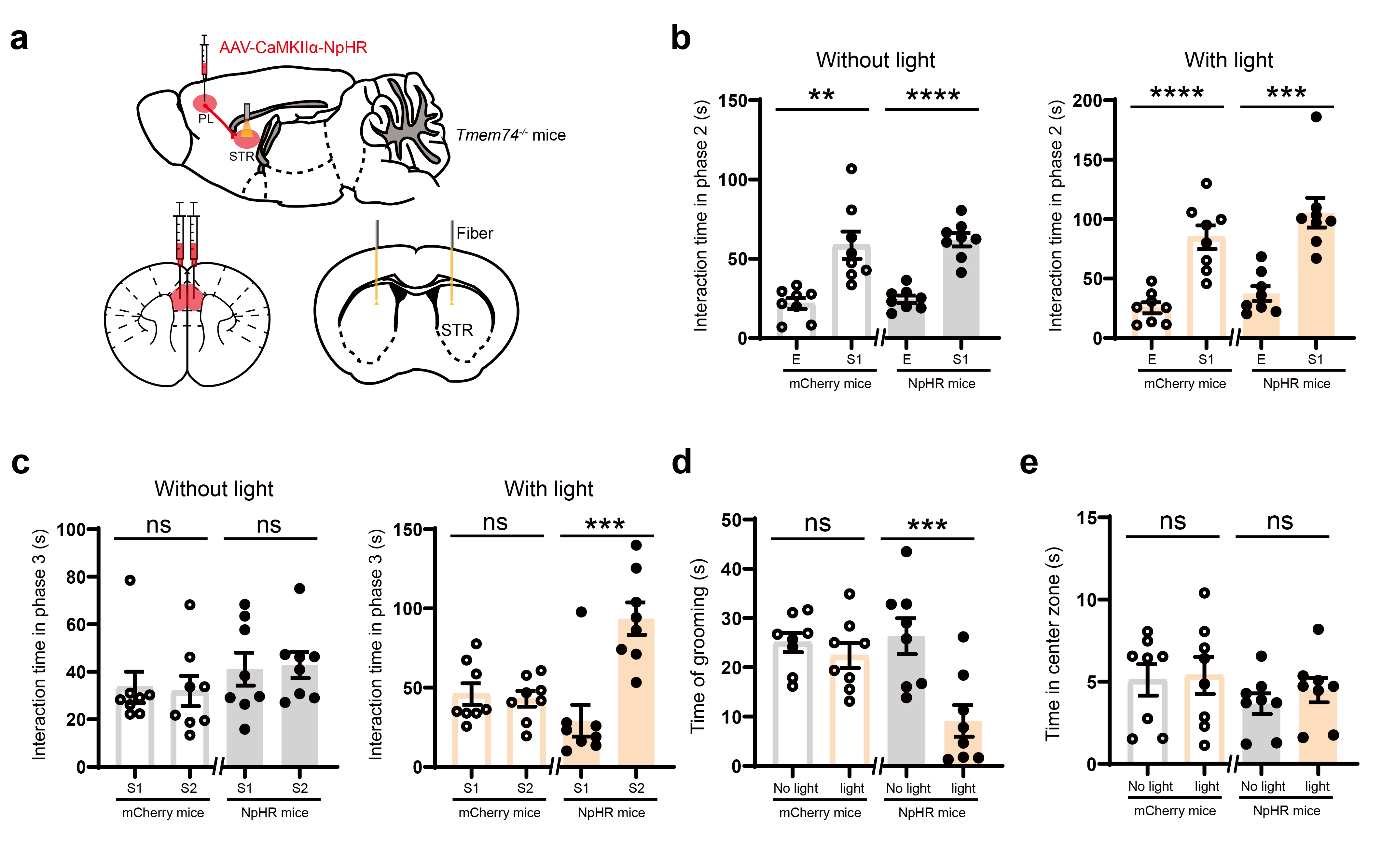


**Supplementary Figure 4 Optogenetic suppression of PL-dSTR rescued autistic-like behavior but not anxiety-like behavior. (a)** Schematic of rAAV2/9-CaMKIIα- mCherry-WPRE (AAV-CaMKIIα-mCherry) or rAAV2/9-CaMKIIα-eNpHR3.0-mCherry-WPRE (AAV- CaMKIIα-NpHR-mCherry) injection in PL and optical fiber placement targeting dSTR terminals. **(b, c)** The interaction time in three-chamber test during phase 2 (**b**) and phase 3 (**c**) with or without yellow light stimulation. **(d)** The time of grooming with or without yellow light stimulation. **(e)** Quantitation of the time in center zone in open field test with or without yellow light stimulation. *Tmem74^-/-^* mice + AAV-CaMKIIα-mCherry: n=8; *Tmem74^-/-^* mice+ AAV- CaMKIIα-NpHR-mCherry: n=8. Data were presented as means ± SEM. ***P* < 0.01, ****P* < 0.001; *****P* < 0.0001; ns, not significant. Unpaired two-tailed Student’s t test for **b** and **c;** Paired two-tailed Student’s t test for **d** and **e.**


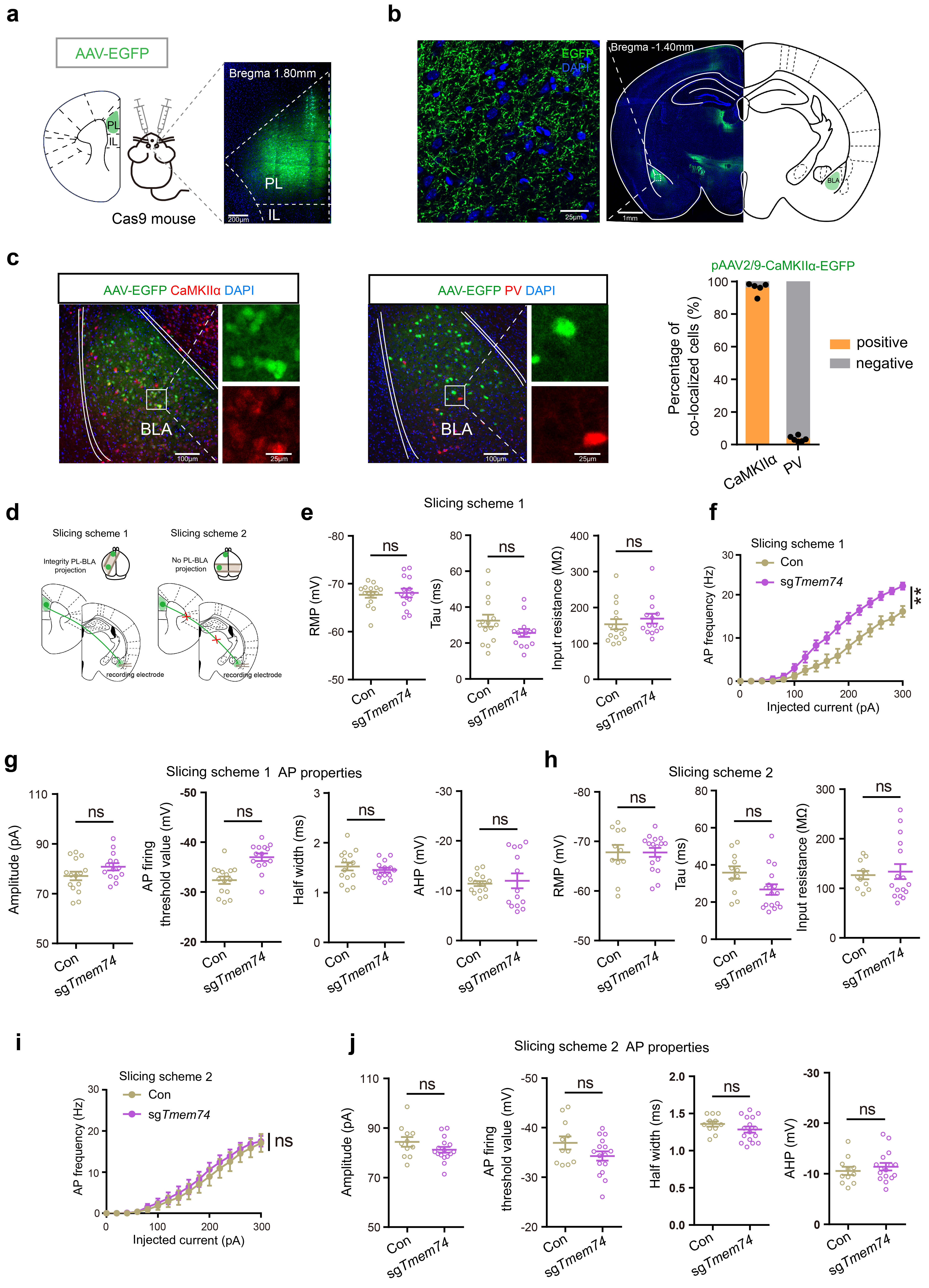


**Supplementary Figure 5 PL projecting to BLA pyramidal neuronal circuits mediated PL regional TMEM74 loss-induced anxiety-like behavior.** (**a**) Left: Diagram of PL injection of pAAV2/9-CaMKIIα-EGFP-2A-MSC-3FLAG (AAV-EGFP) to Cas9 mice. Right: the expression of EGFP in PL region of Cas9 mice. (**b**) A coronal section of EGFP labeled neurons in BLA. (**c**) Representative images and quantification of EGFP-positive and CaMKIIα-positive neurons (95.84%) or PV-positive neurons (3.49%) in BLA. (**d**) Schematic diagram of brain slicing schemes of *ex vivo* electrophysiological recording. (**e-g**) Quantitation of electrophysiological properties of BLA pyramidal neurons in brain slicing scheme 1 (n=15 cells from 3 control mice, n=14 cells from 3 AAV-sg*Tmem74*-injected mice). (**e**) Quantitation of membrane properties (RMP, Tau and Rin) of BLA pyramidal neurons in brain slicing scheme 1. (**f**) Quantification of AP frequency of pyramidal neurons in BLA in the scheme 1. (**g**) Quantitation of AP properties of BLA pyramidal neurons in brain slicing scheme 1. (**h-j**) Quantitation of electrophysiological properties of BLA pyramidal neurons in brain slicing scheme 2 (n=11 cells from 3 control mice, n=16 cells from 3 AAV-sg*Tmem74*-injected mice). (**h**) Quantitation of membrane properties (RMP, Tau and Rin) of BLA pyramidal neurons in brain slicing scheme 2. (**i**) Quantification of AP frequency of pyramidal neurons in BLA in the scheme 2. (**j**) Quantitation of AP properties of BLA pyramidal neurons in brain slicing scheme 2. Data were presented as means ± SEM. ***P* < 0.01; ns, not significant. Unpaired two-tailed Student’s t test for **e**, **g**, **h** and **j**; Two-way ANOVA followed by Sidak’s post hoc test for **f** and **i.**


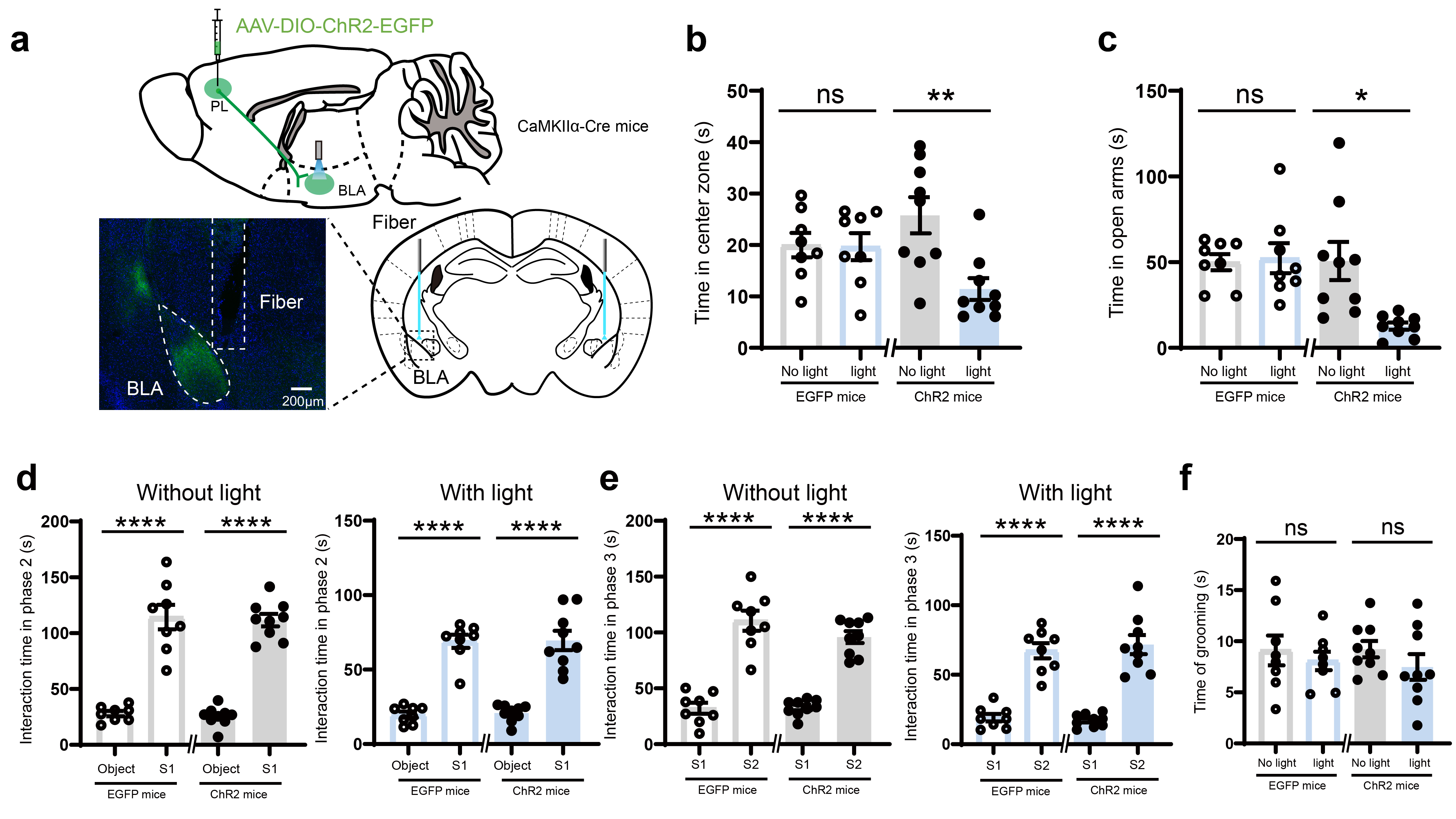


**Supplementary Figure 6 Optogenetic activation of PL-BLA led to anxiety-like behavior but not autistic-like behavior.** (**a**) Schematic and histology of pAAV2/9-EF1α-DIO-EGFP-WPRE (AAV-DIO-EGFP) or pAAV2/9-EF1α-DIO-hChR2 (H134R)-EGFP-WPRE (AAV-DIO-ChR2-EGFP) injection into PL and optical cannula implantation in the bilateral BLA. (**b, c**) The open field test (**b**) and elevated plus maze test (**c**) were performed. (**d**-**f**) The three-chamber test (**d**, **e**) and grooming test (**f**) were performed. CaMKIIα-Cre mice + AAV-DIO-EGFP: n= 8; CaMKIIα-Cre mice + AAV-DIO-ChR2-EGFP: n=9. Data were presented as means ± SEM. **P* < 0.05, ***P* < 0.01, *****P* < 0.0001; ns, not significant. Paired two-tailed Student’s t test for **b, c** and **f**; Unpaired two-tailed Student’s t test for **d** and **e**.


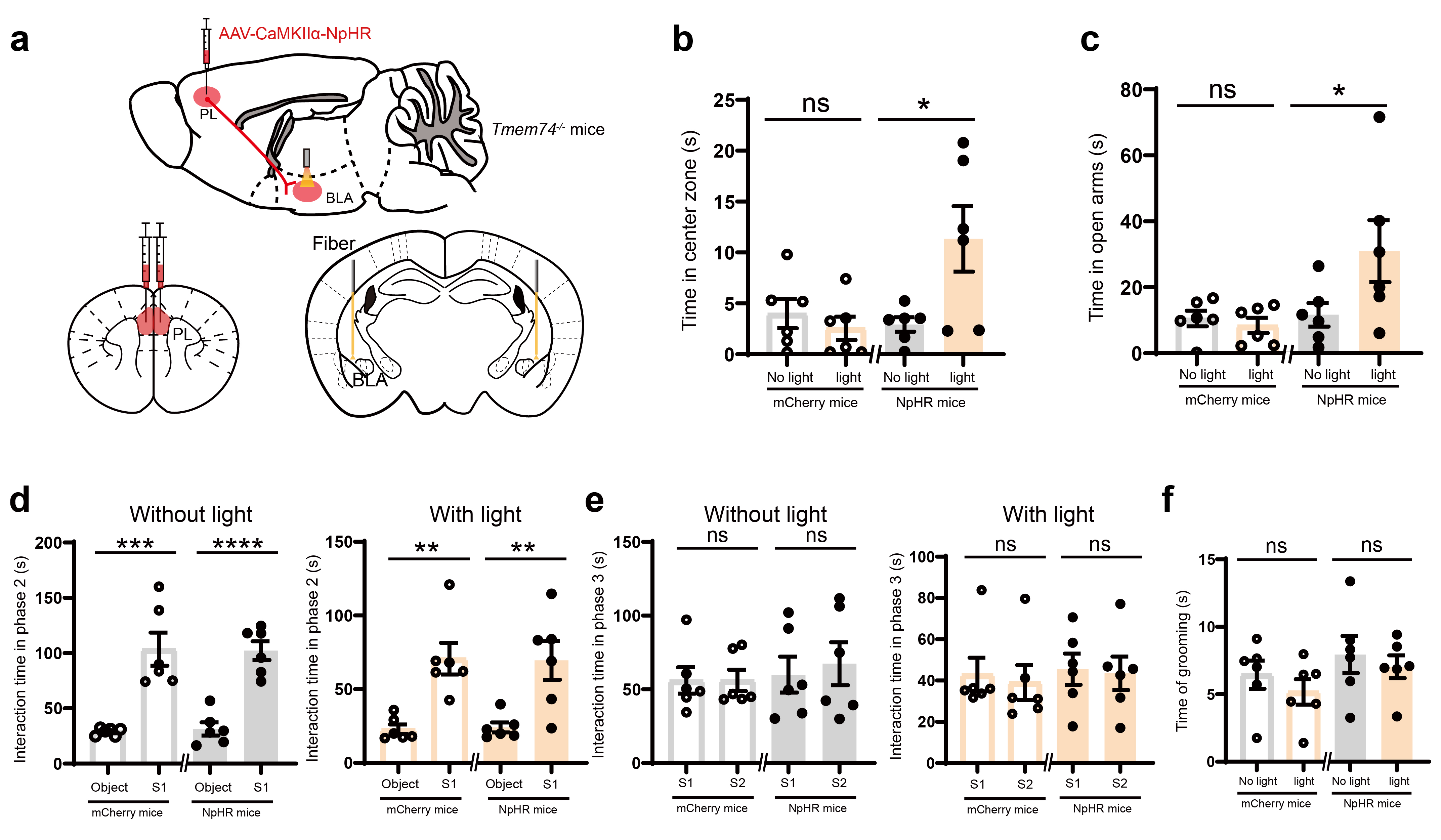


**Supplementary Figure 7 Optogenetic suppression of PL-BLA rescued anxiety-like behavior but not autistic-like behavior. (a)** Schematic of rAAV2/9-CaMKIIα- mCherry-WPRE (AAV-CaMKIIα-mCherry) or rAAV2/9-CaMKIIα-eNpHR3.0-mCherry-WPRE (AAV- CaMKIIα-NpHR-mCherry) injection in PL and optical fiber placement targeting BLA terminals. (**b, c**) The open field test (**b**) and elevated plus maze test (**c**) were adopted. (**d**-**f**) The three-chamber test (**d**, **e**) and grooming test (**f**) were adopted. *Tmem74^-/-^* mice+ AAV-CaMKIIα-mCherry: n=6; *Tmem74^-/-^* mice+ AAV- CaMKIIα-NpHR-mCherry: n=6. Data were presented as means ± SEM. **P* < 0.05, ***P* < 0.01, ****P* < 0.001; *****P* < 0.0001; ns, not significant. Paired two-tailed Student’s t test for **b, c** and **f**; Unpaired two-tailed Student’s t test for **d** and **e**.


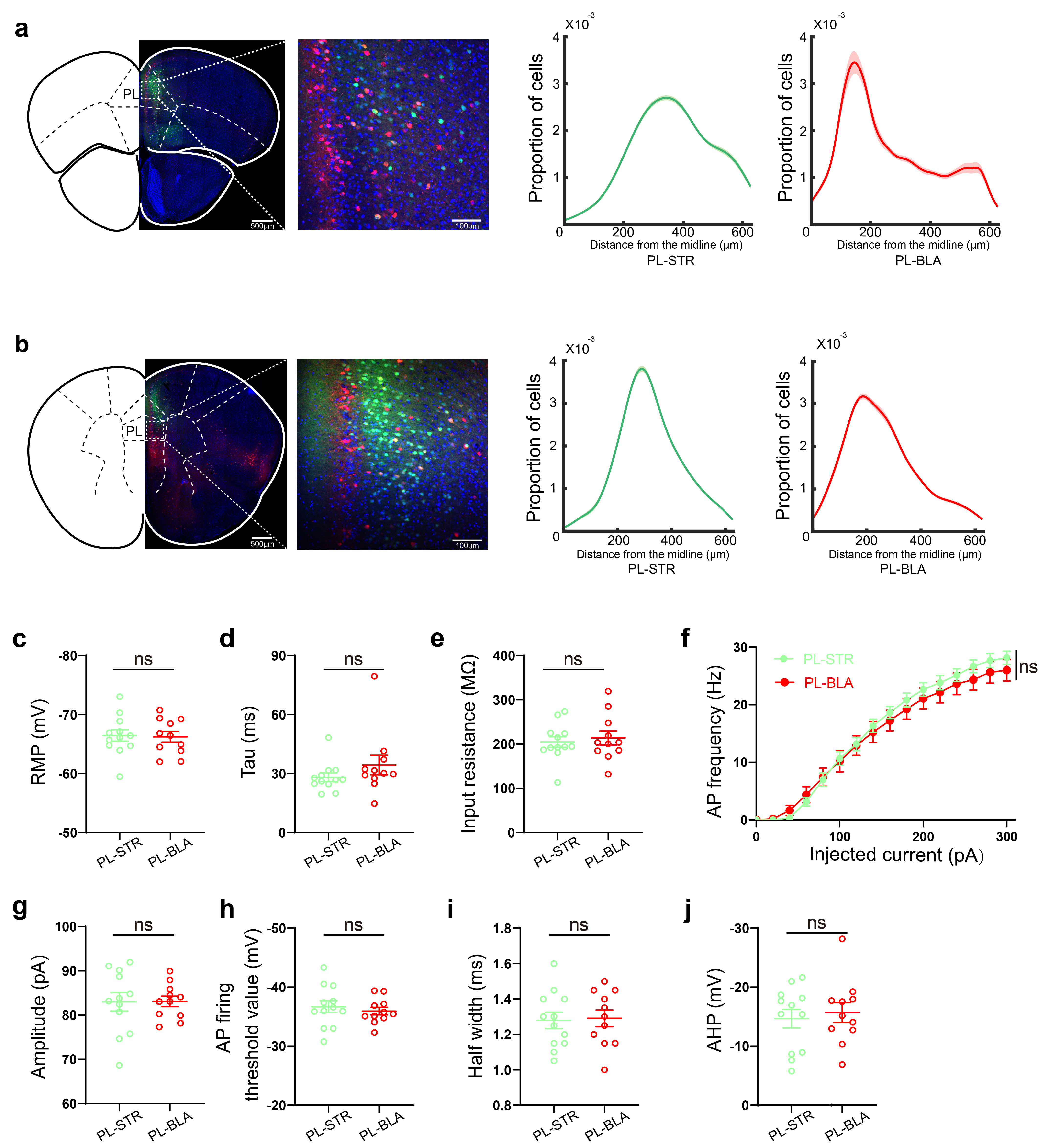


**Supplementary Figure 8 Anatomically distinct subpopulations of PL Neurons project to the STR and BLA.** (**a, b**) Left: A coronal brain slice (**a**, 2.6 mm anterior bregma; **b**, 1.8 mm anterior bregma) of PL neurons in CaMKIIα-Cre mice after rAAV-DIO-EGFP injection into dSTR and rAAV-DIO-mCherry injection into BLA. Right: Quantitation of the medial/lateral distribution of PL pyramidal neurons labeled with green from dSTR injections and red from BLA injections. Shading denotes SEM, Kolmogorov-Smirnov test to compare medial/lateral pyramidal neurons distribution of the 2 populations in a pairwise manner, p=0.0012 (**a**); p=0.1034 (**b**). (**c-j**) The membrane properties and AP properties of EGFP- and mCherry-positvie pyramidal neurons in PL of CaMKIIα-Cre mice. RMP (**c**), Tau (**d**) and input resistance (**e**), AP frequency (**f**), AP amplitude (**g**), AP firing threshold (**h**), AP half-width (**i**) and AHP (**j**)., n=12 cells from PL-STR of 3 mice; n=11 cells from PL-BLA of 3 mice. Data were presented as means ± SEM. **P* < 0.05, ns, not significant. Unpaired two-tailed Student’s t test for **c**-**e** and **g**-**j**; Two-way ANOVA followed by Bonferroni’s post hoc test for **f**.


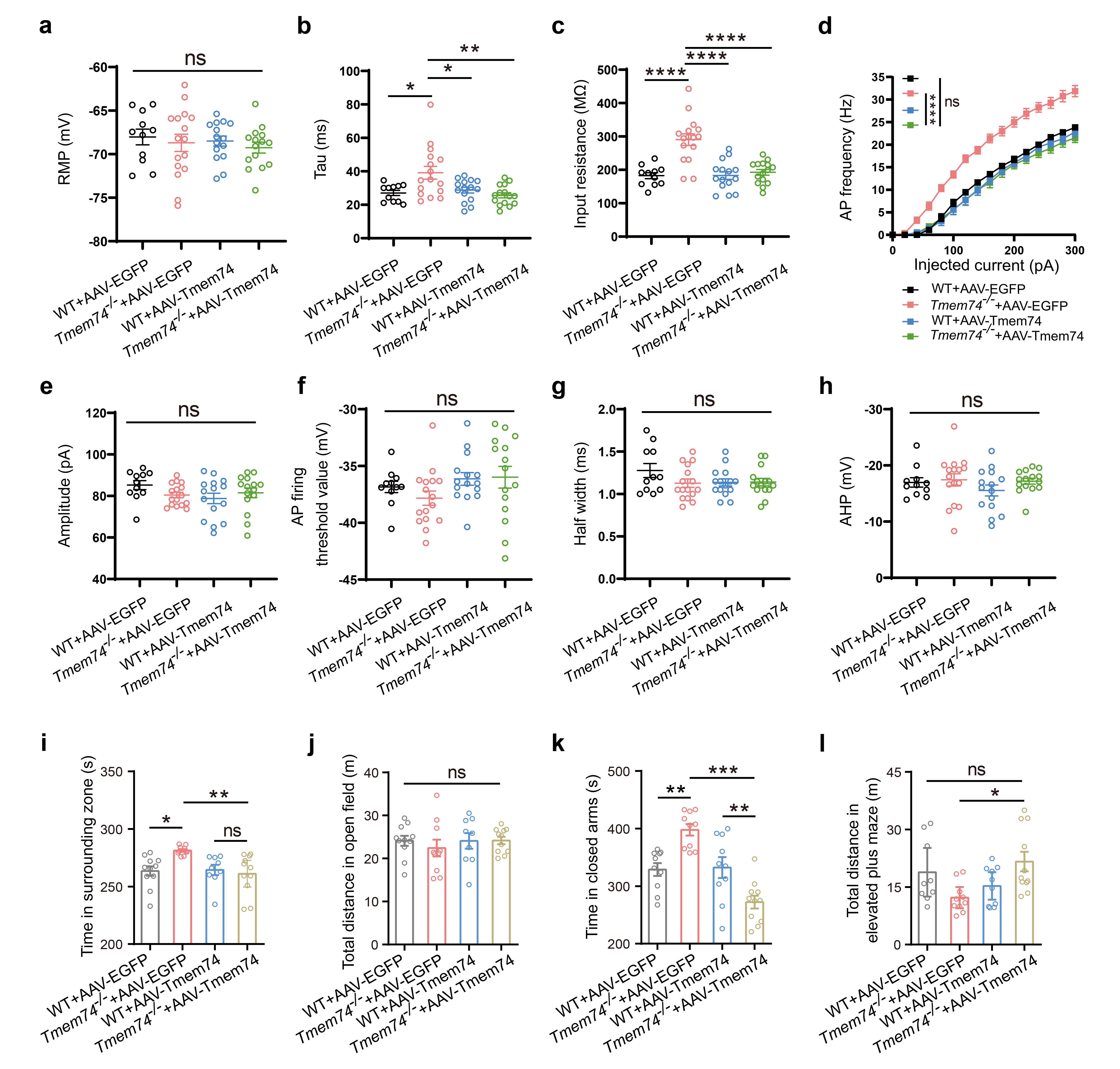


**Supplementary Figure 9 Enrichment of TMEM74 in PL pyramidal neurons rescued abnormal electrophysiological characteristics and anxiety-like behaviors.** (**a-c**) Quantification of RMP (**a**), Tau (**b**) and Rin (**c**) of membrane properties in PL pyramidal neurons under whole-cell recording (n=11 cells from 3 WT+AAV-EGFP mice, n=16 cells from 3 *Tmem74^-/-^*+AAV-EGFP mice, n=15 cells from 3 WT+AAV-Tmem74 mice, n=15 cells from 3 *Tmem74^-/-^*+AAV-Tmem74 mice). (**d-h**) Quantification of the AP frequency (**d**) by current injections from 0 to 300 pA (stepped by 20 pA) and AP properties (**e-h**) of PL pyramidal neurons (n=11 cells from 3 WT+AAV-EGFP mice, n=16 cells from 3 *Tmem74^-/-^*+AAV-EGFP mice, n=15 cells from 3 WT+AAV-Tmem74 mice, n=15 cells from 3 *Tmem74^-/-^*+AAV-Tmem74 mice). AP amplitude (**e**), AP firing threshold (**f**), AP half-width (**g**) and AHP (**h**). (**i-l)** The open field test and elevated plus maze test were performed. Quantitation of the time in corners (**i**) and total distances (**j**) in the open field test, and the time in closed arms (**k**) and total distances (**l**) in elevated plus maze test with the restoration of TMEM74 in PL (n=11 WT+AAV-EGFP mice, n=10 *Tmem74^-/-^*+AAV-EGFP mice, n=9 WT+AAV-Tmem74 mice, n=11 *Tmem74^-/-^*+AAV-Tmem74 mice). Data were presented as means ± SEM. **P* < 0.05, ***P* < 0.01, ****P* < 0.001, *****P* < 0.0001; ns, not significant. One-way ANOVA followed by Tukeyʼs post hoc test was used for **a-c** and **e-l**; Two-way ANOVA followed by Tukeyʼs post hoc test was used for **d**.
